# Reconciling policy instruments with drivers of deforestation and forest degradation: cross-scale analysis of stakeholder perceptions in tropical countries

**DOI:** 10.1038/s41598-023-29417-y

**Published:** 2023-02-07

**Authors:** Rubén Ferrer Velasco, Melvin Lippe, Richard Fischer, Bolier Torres, Fabián Tamayo, Felix Kanungwe Kalaba, Humphrey Kaoma, Leonida Bugayong, Sven Günter

**Affiliations:** 1 Ecosystem Dynamics and Forest Management Group, School of Life Sciences, Technical University of Munich (TUM), 85354 Freising, Germany; 2 Institute of Forestry, Johann Heinrich von Thünen Institute, 21031 Hamburg, Germany; 3 Life Sciences Department, Universidad Estatal Amazónica (UEA), 160101 Puyo, Ecuador; 4 School of Natural Resources, Copperbelt University, 21692 Kitwe, Zambia; 5 Forestry Development Center, College of Forestry and Natural Resources, University of the Philippines Los Baños, 4031 Laguna, Philippines

**Keywords:** Forestry, Psychology and behaviour, Sustainability, Tropical ecology, Environmental impact

## Abstract

Cross-scale studies combining information on policy instruments and on drivers of deforestation and forest degradation are key to design and implement effective forest protection measures. We investigated the scale and country dependency of stakeholder perceptions about future threats to tropical forests (e.g. agriculture, logging, woodfuel) and preferred policy instruments (e.g. reforestation, protected areas, combat illegal logging), by interviewing 224 representatives of forest-related institutions. We conducted analysis of variance and principal component analysis for eighteen variables across three countries (Zambia, Ecuador and the Philippines) and four spatial levels (from international to local). We found that the overall alertness about commercial drivers and the confidence in policy instruments are significantly lower at subnational levels and also in Zambia. Stakeholder expectations about the most important drivers and the most effective policies in the coming decade follow regional narratives, suggesting that there are no one-size-fits-all solutions in international forest policy. However, we found an unexpected consensus across scales, indicating potential for collaboration between institutions operating at different geographical levels. Overall, agriculture remains the driver with the highest expected influence (43%), while a strong favoritism for reforestation and forest restoration (38%) suggests a paradigm shift from protected areas to a stronger focus on integrative approaches.

## Introduction

Although current tropical deforestation rates (9.3 million ha/yr between 2015 and 2020) have slowed down when compared to previous decades (e.g. 13.8 million ha/yr between 1990 and 2000)^1^, tropical forests still account for more than 90% of the total forest loss worldwide. This deforestation trend, linked to processes of forest fragmentation and degradation^2,3^, poses a threat to the multiple ecosystem services of tropical forests, which are essential for human well-being^4,5^. This wide range of ecosystem services provided by tropical forests has profound impacts both locally and globally (e.g. on weather patterns, water cycle, natural catastrophes, biodiversity or food and human health)^5,6^.

The drivers behind this trend have been well studied^7^. Already in the early 2000’s forest scholars and practitioners identified pantropical patterns and distinguished between proximate and other underlying driving forces^8–10^. This classification had been introduced in the early nineties in the context of anthropogenic global environmental change^11,12^ and it has been widely accepted and used^7,13–15^. More recently, further investigations have used econometric and spatial analyses and survey or remote sensing data to quantify and characterize the main direct causes of deforestation and forest degradation in the tropics^16–20^. These drivers are complex and region-dependent^16,17,20^, but they are mostly related to land-use and anthropogenic pressure^19,20^: e.g. expansion of commercial and subsistence agriculture, legal and illegal logging, fuelwood collection, charcoal production, expansion of timber plantations, oil extraction, surface mining, urban and infrastructure, and wildfires or other natural disasters.

As a response to these threats, an increasing number and variety of policy instruments for the protection and conservation of forests have been implemented in tropical landscapes over the last decades^21,22^. Some examples include: protected areas, reforestation activities, measures against logging or land tenure reforms. Conventionally, such policy instruments are classified into regulatory (command and control), economic and informational (sermons), while they imply a set of enabling, positive (carrots) or negative (sticks) incentives and regulations^21–23^. Nevertheless, the effectiveness of these instruments is very context-dependent and usually well-designed mixes of policies are recommended^21,22,24^. More recently, market-based and demand-led policy instruments involving public and private actors (e.g. payments for ecosystem services [PESs] such as the “Reducing emissions from deforestation and forest degradation” program [REDD +], certification or supply-chain initiatives), have shown their potential (and limitations) to be effective in halting deforestation with favorable institutional and governance contexts^21,25,26^.

Our work addresses two main gaps in existing empirical research. First, there is a lack of pantropical studies which combine both information on drivers of deforestation and the suitability or effectiveness of different policy instruments. Improving the knowledge about such interrelations is important, because the design and implementation of effective forest protection measures requires addressing the specific forces that drive forest loss in a particular context. Most of the previous literature focuses on single countries and circumstances^27–29^ or on specific drivers and/or policy options^30–33^. However, broader approaches can orientate us towards more general conclusions and provide useful insights on the links between the main threats and solutions related to tropical deforestation. Secondly, pantropical cross-scale studies about the drivers of deforestation and/or policy instruments are even scarcer (i.e. across spatial levels related to interconnected geographical jurisdictions, from global to local: e.g. international, national, provinces, districts, municipalities…). Despite previous studies examining the cross-scale effects of tropical deforestation^34,35^, research has largely focused on single countries^36–40^. Deriving meaningful empirical findings from cross-scale information is a challenging task, which implies overcoming a number of mismatches between data of varying nature, quality and very different acquisition methods^20,32^. For instance, many relevant statistics are, if available, collected at provincial or national levels (e.g. land cover maps, commodity production, exports or agricultural yields). Thus, the majority of pantropical studies still work with national or regional aggregations^18,41,42^. The information at local levels usually relies on perceptions and on disaggregated estimations^32,43,44^. Nevertheless, integrated analyses that consider the circumstances of each jurisdiction across the spatial scale where both drivers of deforestation and policy instruments act, can support more comprehensive deliberations over the appropriate mix of policy tools and strategies needed to successfully combat deforestation^24^.

In our work, we aim to shed some light on the abovementioned gaps by answering the following research questions:

- Are perceptions of relevant key informants and stakeholders in the tropics the same across countries (Zambia, Ecuador and the Philippines) and across scales (spatial levels or geographical jurisdictions, i.e. international, national, regional [subnational] and local) regarding:
  - Future drivers of deforestation and forest degradation and
  - Preferred policy instruments for forest protection?

To address these questions, we use data from a questionnaire conducted between 2018 and 2019 with 224 representatives of forest-related institutions in Zambia, Ecuador and the Philippines. We analyze responses across the three countries and four spatial levels (geographical jurisdictions from international to local), by conducting analysis of variance (ANOVA) and principal component analysis (PCA) for eighteen relevant variables. The studied variables are indicators of the stakeholders’ general perception (i.e. alertness about commercial/subsistence drivers and confidence in policy measures), as well as expected relative importance and effectiveness of specific cross-country driver and policy instrument categories, respectively.

We hypothesize that clear country and scale dependencies can be identified among the interviewed stakeholders, as both drivers and policy instruments vary strongly across regions and scales^20^. For instance, a higher prevalence of commodity-driven deforestation over shifting agriculture has been identified in South America and South East Asia, when compared to Sub-Saharan Africa^16,17,33^. Similarly, certain policy instruments, such as PES schemes, count with a longer history of implementation in specific regions^45^, with most of the research on their effectiveness being conducted in South America^22^. Another current example of such regional differences is the prioritization of Africa within the Bonn Challenge, where 130 million hectares of degraded forest have been pledged to be restored by 2030 (roughly 20% of total forest extent in Africa), in contrast to 47 million hectares in Latin America and the 29 million hectares in Asia and the Pacific (5 and 4% of total forest area, respectively)^46^. Likewise, we expect cross-scale differences because deforestation drivers and policy instruments act at different spatial levels or interconnected geographical jurisdictions, from global (e.g. international trade of commodities or internationally-funded protection schemes) and national (e.g., planning of infrastructure development or national protected areas), to subnational and local (e.g. subsistence agriculture and forest resource extraction or community-based forestry). A further reason to expect country and scale dependencies in our findings, is the existence of different stakeholder configurations in each context. Stakeholders have specific responsibilities or management roles and perceptions based on particular interests and experiences of success or failure in the past. For instance, national stakeholders (e.g. ministry representatives) are usually involved in the design of de jure practices, considering the threats to forest and potential protection mechanisms from a broader perspective. In contrast, local stakeholders (e.g. municipality officers) are typically closer to the implementation on the field and de facto practices^47–49^.

Our hypothesis can be underpinned theoretically by some of the frameworks used in research on forest-related conflicts^50^. Forest conflicts have been defined as “differing views of reality and underlying cultural biases”^51^ or “incompatibility of interests over the same territory or resource”^52^. Such conflicts, not necessarily involving dramatic confrontations or negative changes, are intrinsic to forest governance/management and happen at a range of geographical levels^53^. The theoretical approaches of literature^50^ are typically classified into structural–functional (i.e. related to economic and political distribution of power over forest resources), neo-institutionalism (i.e. considering the influence of formal and informal rules on the behavior of individuals and groups or public/private actors) and perceptional–ideational (i.e. contrasting storylines, narratives, values-beliefs, discourses or frames). Our work is a clear example of how these frameworks overlap in practice and how they can help to explain the country and scale dependencies of stakeholder perceptions as described in the previous paragraph.

## Results

### Sample and answers

Our sample included a comparable number of observations per country, ranging from 66 and 73 key informant questionnaires in Ecuador and Zambia respectively, to 85 in the Philippines (Table 1). Most of the institutions of the interviewed stakeholders belonged to the national and regional levels (82 and 72 respectively), whereas 52 were local institutions. The international level was represented by 18 observations. The distribution across spatial levels was similar between countries, but Zambia had a slightly higher share of regional institutions (48%, versus 27% and 22% in Ecuador and the Philippines, respectively). Ecuador and the Philippines had a relatively larger proportion of national institutions (28% and 38%, respectively, versus 16% in Zambia). Further details about the respondents and their institutions constituting our sample are included in Supplementary Table S1.

**Table 1:** Number of interviews conducted per country and spatial level of the participants’ institutions.

		Spatial level				Total
		International	National	Regional	Local	
Country	Zambia	6	16	35	16	73
	Ecuador	7	28	18	13	66
	Philippines	5	38	19	23	85
Total		18	82	72	52	224

We include a comprehensive list of the answers provided by the respondents in each country, grouped by driver and policy instrument categories, as Supplementary Tables S2 and S3 online. Additionally, we summarize the Likert scores and ranking answers grouped by country and spatial level (see Supplementary Figs. S1–S8). Details on the descriptive statistics and the distributions of the studied variables can also be found as Supplementary Tables S4 to S7 online. Table 2 lists the eighteen variables included in our study and their definition.

**Table 2:** Variables included in the statistical analysis and their definition (observation unit: respondent).

Variable	Definition/classification
Overall perceptions	
Alertness	Share (%) of total answers on drivers of deforestation with “strong” (4) or “very strong” (5) influence
AlertnessCom	Share (%) of total answers on drivers of deforestation related to commercial economy with “strong” (4) or “very strong” (5) influence
AlertnessSub	Share (%) of total answers on drivers of deforestation related to subsistence economy with “strong” (4) or “very strong” (5) influence
Confidence	Share (%) of total answers on policy instruments with “strong” (4) or “very strong” (5) influence
Expected importance of drivers of deforestation and forest degradation (future 10 years)	
Agriculture	Expected importance (%) of drivers related to expansion of agriculture (includes commercial and subsistence, crops/pastures/agroforestry, shifting cultivation…)
Logging	Expected importance (%) of drivers related to logging and extraction of timber and other forest resources involving tree cutting (both legal/illegal activities)
Woodfuel	Expected importance (%) of drivers related to firewood/woodfuel collection and charcoal production
Oilmining	Expected importance (%) of drivers related to oil and mining activities (e.g. exploration)
Infrastructure	Expected importance (%) of drivers related to expansion of urban areas and infrastructure development (road construction, bridges…)
Plantations	Expected importance (%) of drivers related to the expansion of timber plantations
Naturaldisasters	Expected importance (%) of drivers related to natural disasters (e.g. drought, fires, flooding, landslides, earthquakes…)
Otherdrivers	Expected importance (%) of other drivers mentioned by the participants (mostly underlying drivers, e.g. political and tenure conflicts, lack of education)
Expected effectiveness of policy instruments (future 10 years)	
Reforestation	Expected effectiveness (%) of policy instruments related to reforestation, regrowth of natural forest, passive/active forms of forest restoration or establishment of agroforestry areas
Protectedareas	Expected effectiveness (%) of policy instruments related to protected areas restricting access or use of forest, including state reserves, indigenous or private forests
AntiLogging	Expected effectiveness (%) of policy instruments related to measures against illegal logging, including different forms of banning, moratoriums, stronger controls (e.g. patrolling, rangers, regulating timber exports)
Financialtools	Expected effectiveness (%) of policy instruments related to financial mechanisms, including certification, business-funded incentives or PESs (e.g. REDD +)
Landuserights	Expected effectiveness (%) of policy instruments related to improved and secured land titling, decentralization, local participation, community-based and integrated forest management
Otherpolicies	Expected effectiveness (%) of other policy instruments mentioned by the participants, e.g. improving education, sensitization, promoting alternative livelihood/energy sources, international involvement, better governance, less political interference

### One-way analysis of variance (ANOVA)

Table 3 depicts the results (levels of significance) of the non-parametric one-way ANOVAs for the eighteen studied variables across countries and spatial levels, overall and between groups. In this table and in the following subsections, we will show the results of the non-parametric tests, as we could not demonstrate univariate and multivariate normality of our sample. However, we also conducted parametric tests (with stronger statistical power) as an additional support of the validity of our findings. Thus, for all variables, the significance of the parametric and non-parametric tests was identical, regarding the overall results across countries and spatial levels. Only a few disagreements occurred at the specific group comparisons. On the one hand, the parametric ANOVA (Tukey test) detected statistically significant differences between Ecuador and the Philippines for three variables, and between the national and the local level for one variable. On the other hand, the non-parametric Dunn test detected statistically significant differences between four pair combinations of spatial levels, concerning two variables. The extended results of both parametric and non-parametric analyses are included as Supplementary Tables S8 to S11 online.

**Table 3:** Results of non-parametric ANOVAs for the eighteen studied variables, with overall cross-country and cross- ‘spatial level’ significances (***: < 0.001, **: < 0.01, *: < 0.05, *ns* not significant [> 0.05], underlined: disagreement between parametric and non-parametric methods) and results for group comparisons (*Zmb* Zambia, *Ecu* Ecuador, *Phl* Philippines, *Int* International, *Nat* National, *Reg* Regional, *Loc* Local).

Variables	Across countries				Across spatial levels						
	Country	Zmb-Ecu	Zmb-Phl	Ecu-Phl	Spatial level	Int-Nat	Int-Reg	Int-Loc	Nat-Reg	Nat-Loc	Reg-Loc
Overall perceptions											
Alertness	***	***	***	ns	***	ns	*	**	**	***	ns
AlertnessCom	***	***	***	ns	***	ns	*	**	**	***	ns
AlertnessSub	ns	ns	ns	ns	ns	ns	ns	ns	ns	ns	ns
Confidence	***	***	***	ns	**	ns	ns	ns	**	ns	ns
Expected importance of drivers											
Agriculture	***	**	ns	**	ns	ns	ns	ns	ns	ns	ns
Logging	**	**	**	ns	ns	ns	ns	ns	ns	ns	ns
Woodfuel	***	***	***	ns	**	ns	ns	ns	**	ns	ns
OilMining	***	***	ns	ns	**	ns	ns	ns	ns	**	**
Infrastructure	ns	ns	ns	ns	ns	ns	ns	ns	ns	ns	ns
Plantations	ns	ns	ns	ns	ns	ns	ns	ns	ns	ns	ns
Naturaldisasters	***	ns	***	***	ns	ns	ns	ns	ns	ns	ns
Otherdrivers	**	ns	**	ns	ns	ns	ns	ns	ns	ns	ns
Expected effectiveness of policy instruments											
Reforestation	***	***	ns	**	ns	ns	ns	ns	ns	ns	ns
Protectedareas	***	***	***	ns	ns	ns	ns	ns	ns	ns	ns
AntiLogging	ns	ns	ns	ns	ns	ns	ns	ns	ns	ns	ns
Financialtools	***	***	ns	***	ns	ns	ns	ns	ns	ns	ns
Landuserights	ns	ns	ns	ns	ns	ns	ns	ns	ns	ns	ns
Otherpolicies	***	***	***	ns	***	ns	ns	ns	***	ns	*

#### Overall alertness about drivers and confidence in policy instruments

**Figure 1:**
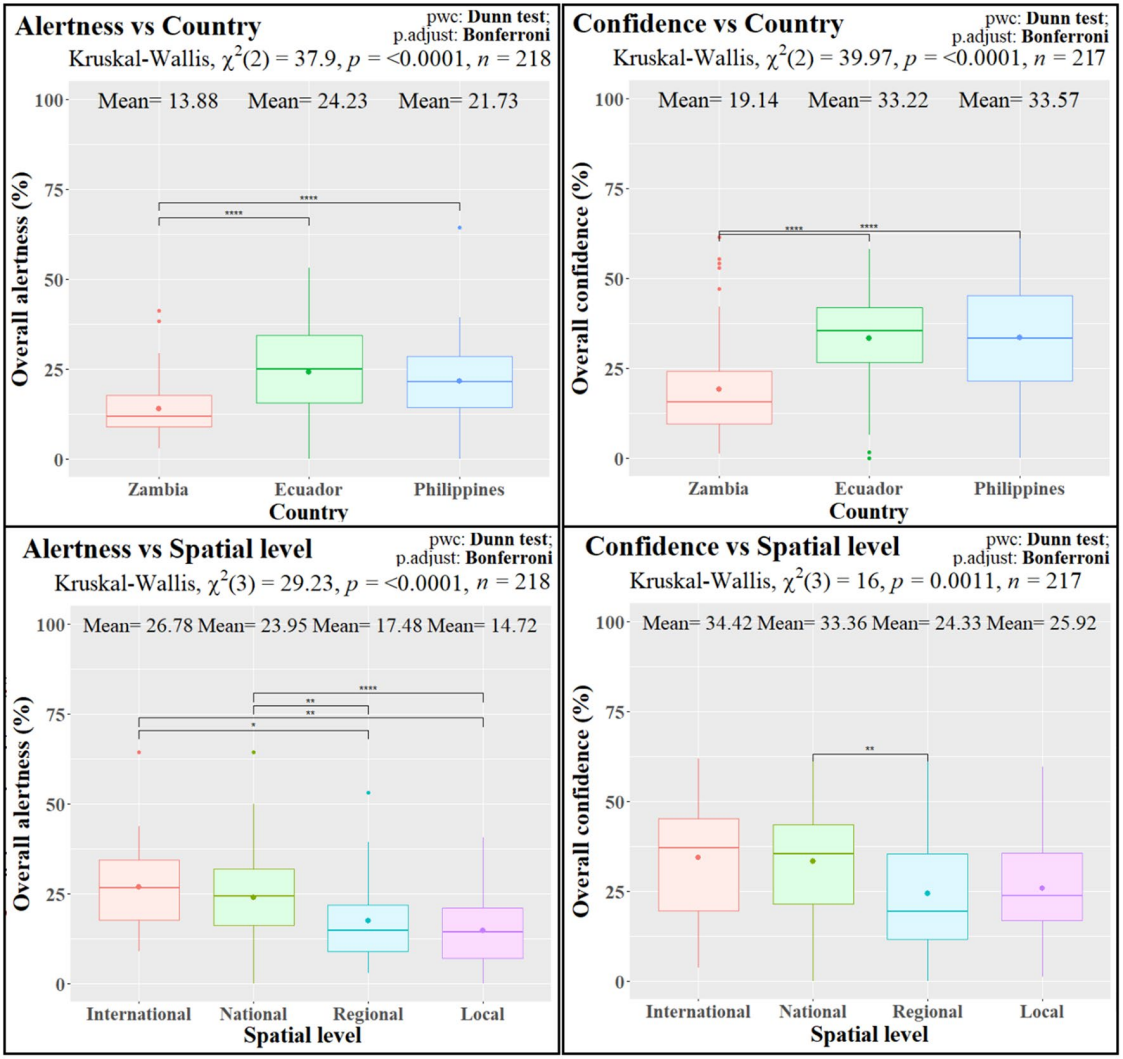
Results of the Kruskal–Wallis and Dunn tests across countries and spatial levels for *Alertness* (overall alertness about deforestation drivers) and *Confidence* (overall confidence in policy instruments). Boxplots including mean values (Mean), chi square statistic (χ^2^), p-values (p) and number of observations (n) of the Kruskal–Wallis tests and p-adjustment (p. adjust) and p-scores (sign, ****: < 0.0001, ***: < 0.001, **: < 0.01, *: < 0.05, *ns* not significant [> 0.05]) for the Dunn pairwise comparisons (pwc).

For both overall alertness about deforestation drivers (*Alertness*) and overall confidence in policy instruments (*Confidence*)*,* we could detect significant differences across countries and spatial levels (Table 3, Fig. 1). In the case of *Alertness*, the differences were related to the drivers connected to the commercial economy (*AlertnessCom*). No significant differences across countries or spatial levels were observed regarding the alertness about drivers linked to subsistence economy (*AlertnessSub*).

When compared to the other two countries, Zambia showed significantly lower *Alertness* (14% average, vs. 24% and 22% in Ecuador and the Philippines, respectively), *AlertnessCom* (12% vs. 35% and 30%) and *Confidence* (19% vs. 33% and 34%). *AlertnessSub* was generally lower than *AlertnessCom* (overall 14% vs. 26%), with the exception of Zambia (16% vs. 12%).

We also observed that *Alertness* and *AlertnessCom* decreased gradually from the international to the local institutions. The average *Alertness* was 27% at the international level, 24% at the national level, 17% at the regional (subnational) level and 15% at the local one. Concerning *AlertnessCom*, the average values for the different spatial levels were 36%, 32%, 22% and 18%, respectively. According to the non-parametric Dunn test, all the differences between groups were significant for both variables, except for the pairs international-national and regional-local. *Confidence* showed a similar decreasing trend, with average values of 34%, 33%, 24% and 26% for the international, national, regional and local levels, respectively. However, we could only demonstrate statistically significant differences between the national and the regional (subnational) level.

#### Expected future importance of drivers of deforestation and forest degradation

**Figure 2:**
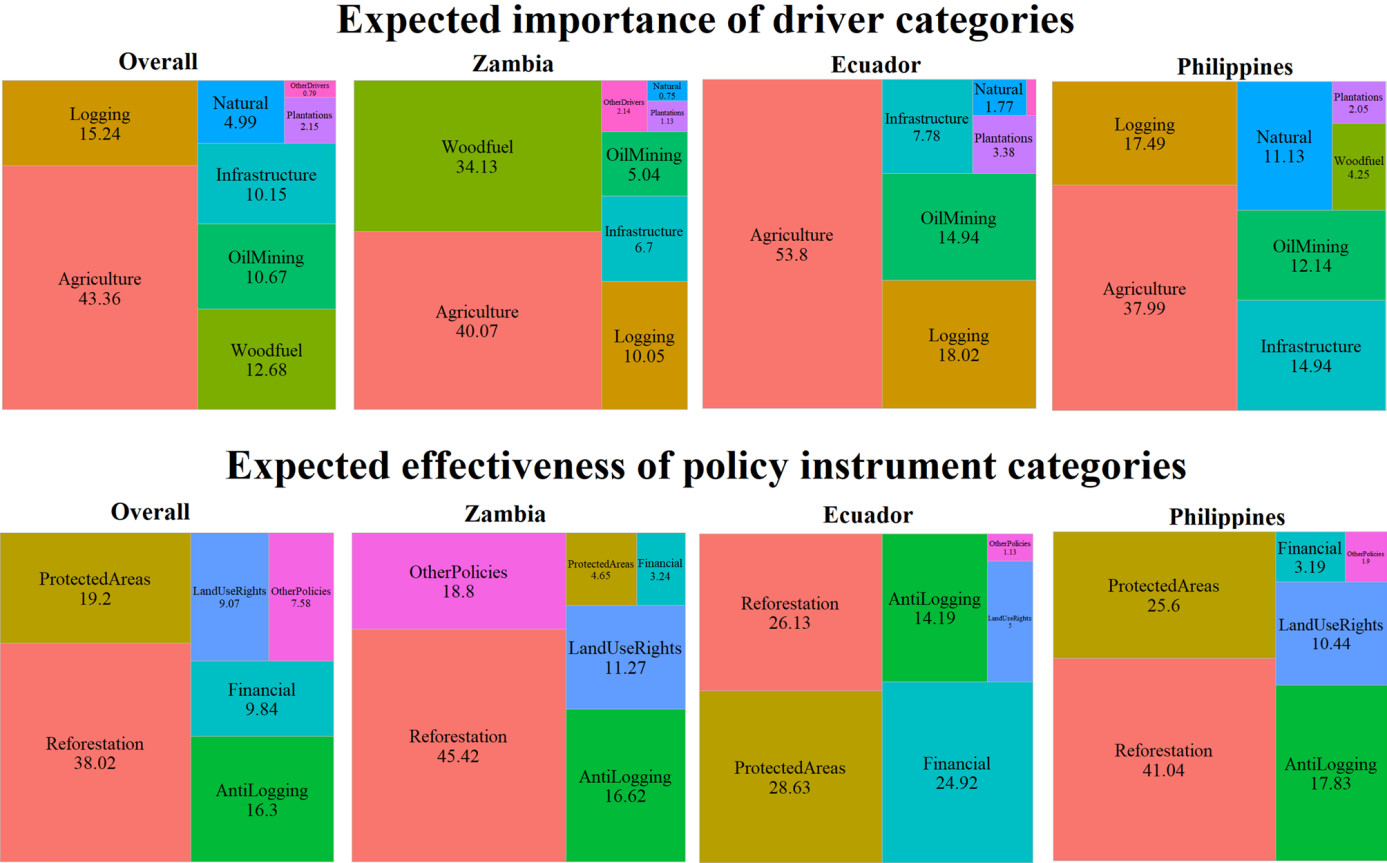
Tree maps representing the overall expected importance of the driver categories and the expected effectiveness of the policy instrument categories in the total sample and in the country subsamples. Natural refers to *NaturalDisasters*, Financial refers to *FinancialTools*.

**Figure 3:**
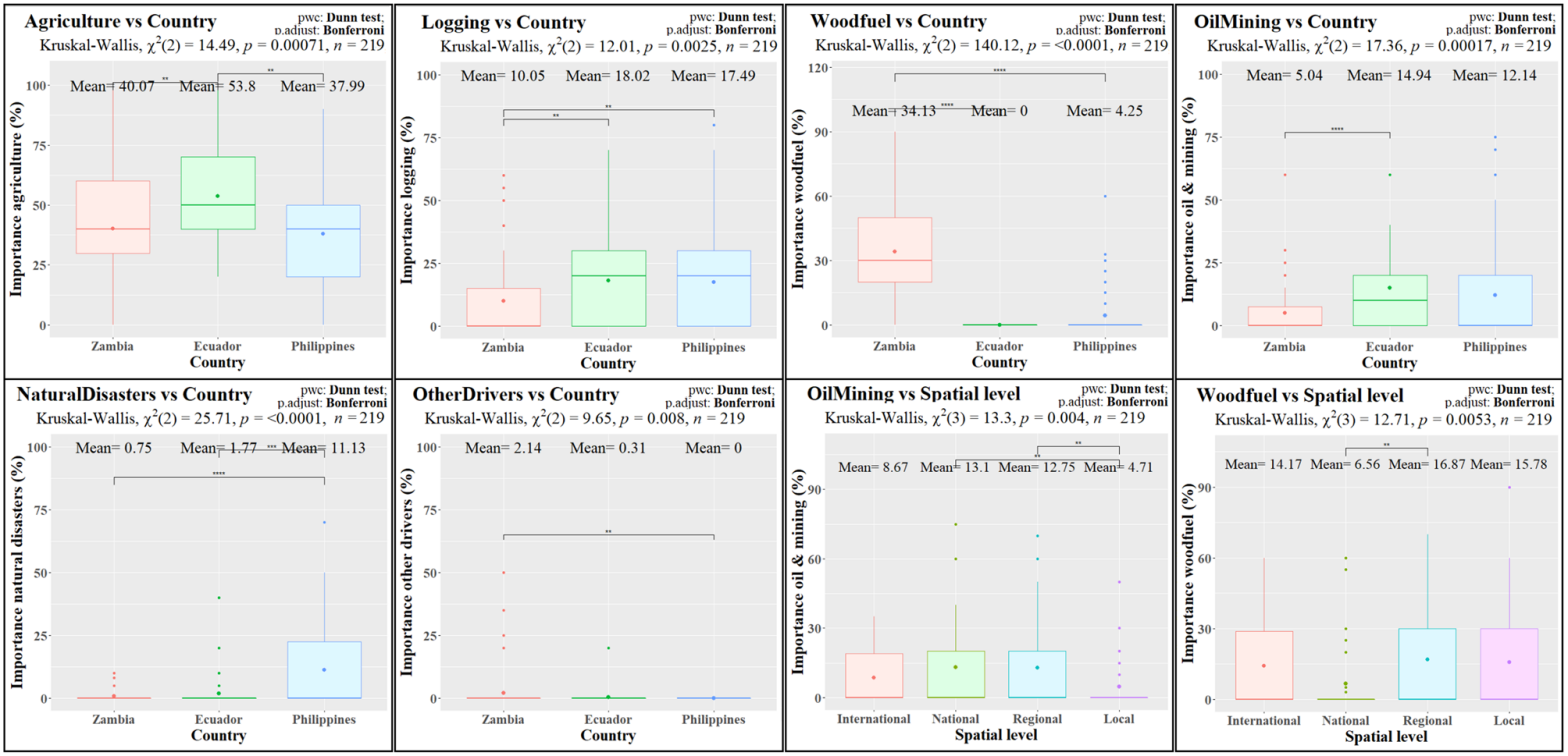
Results of the Kruskal–Wallis and Dunn tests across countries and spatial levels for the variables related to the expected importance of driver categories. Only tests with statistically significant results are shown. Boxplots including mean values (Mean), chi square statistic (χ^2^), p-values (p) and number of observations (n) of the Kruskal–Wallis tests and p-adjustment (p.adjust) and p-scores (sign, ****: < 0.0001, ***: < 0.001, **: < 0.01, *: < 0.05, *ns* not significant [> 0.05]) for the Dunn pairwise comparisons (pwc).

*Agriculture* was expected to be the most important driver category in the three countries: i.e. overall, 43% importance (Fig. 2). In Ecuador (Fig. 3 and Table 3), the importance of *Agriculture* was significantly higher (54%) than in Zambia (40%) and the Philippines (38%). *Logging* was identified as the second most important driver overall (15% importance), but with significantly lower relative importance in Zambia (10%) than in Ecuador (18%) and the Philippines (17%). Instead, Zambia had significantly higher importance (34%) for *Woodfuel*, which stayed below 5% in the Philippines and was not mentioned by the participants in Ecuador. The rest of drivers showed lower relevance overall, but still with some significant differences between countries. For instance, *OilMining* was significantly higher in Ecuador (15% importance), while *NaturalDisasters* was significantly more important in the Philippines (11% vs. 1% and 2% in Zambia and Ecuador, respectively). The Philippines also showed higher results for *Infrastructure* (15% vs. 7% and 8%), although these differences were not statistically significant. In Zambia, the importance of *OtherDrivers* was also significantly higher but still relatively low (2%). Finally, no cross-country differences were detected for the generally low importance (2% overall) of *Plantations*.

Across spatial levels, however, we only detected two statistically significant differences (Fig. 3 and Table 3). First, we found significantly lower importance of *OilMining* at local levels (5%) when compared to national (13%) and regional (13%) stakeholders. Second, *Woodfuel* was significantly more important at regional (subnational) levels (17%) than at national levels (7%).

#### Expected future effectiveness of policy instruments

**Figure 4:**
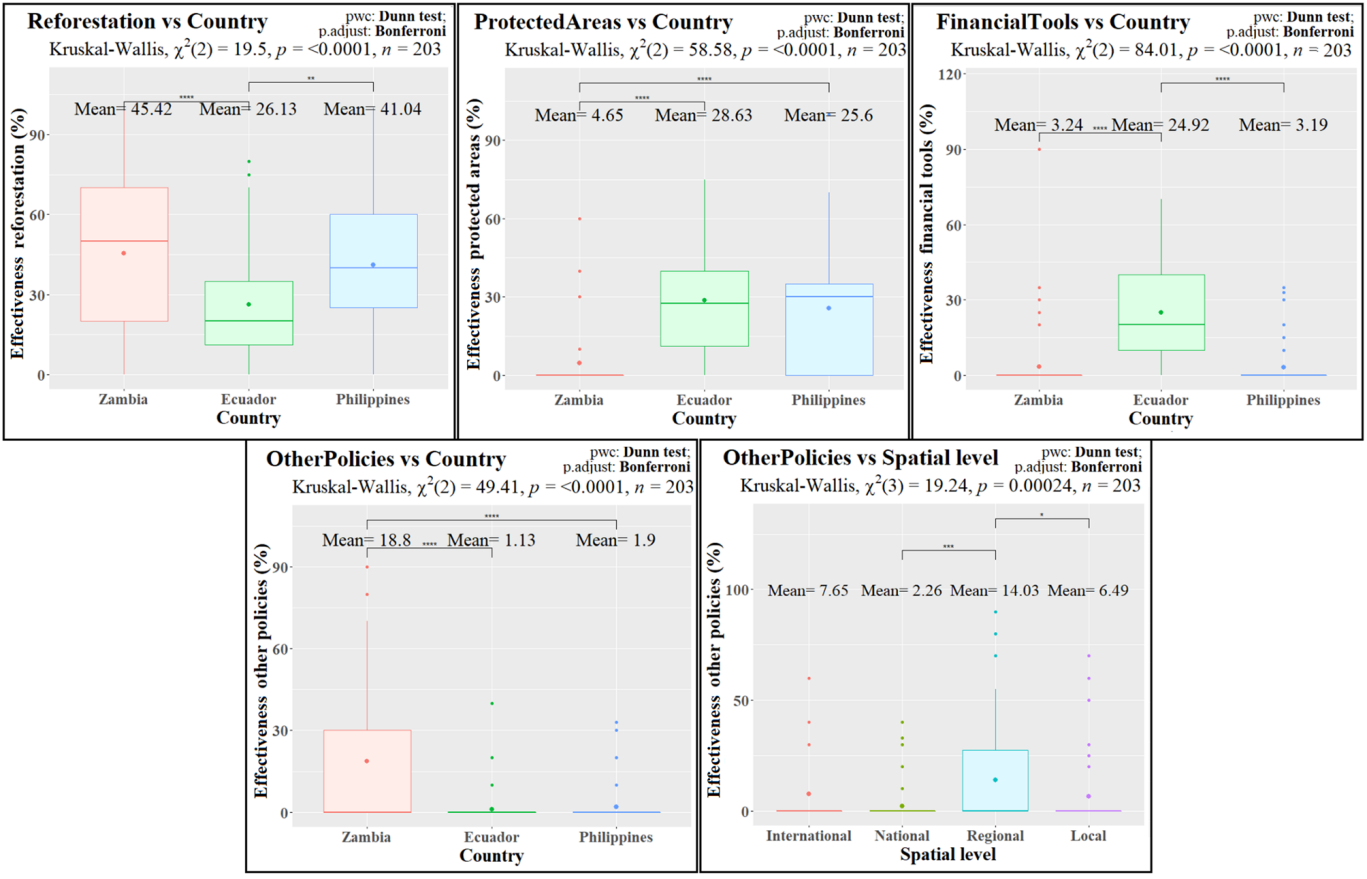
Results of the Kruskal–Wallis and Dunn tests across countries and spatial levels for the variables related to the expected effectiveness of policy instrument categories. Only tests with statistically significant results are shown. Boxplots including mean values (Mean), chi square statistic (χ^2^), p-values (p) and number of observations (n) of the Kruskal–Wallis tests and p-adjustment (p. adjust) and p-scores (sign, ****: < 0.0001, ***: < 0.001, **: < 0.01, *: < 0.05, *ns* not significant [> 0.05]) for the Dunn pairwise comparisons (pwc).

We could further detect country dependencies on the expected effectiveness of policy instruments (Fig. 4 and Table 3). *Reforestation* (Fig. 2) was the favorite category overall (38%), with statistically higher effectiveness assigned by the stakeholders in Zambia (45%) and the Philippines (41%) than in Ecuador (26%). The second most effective instrument was *ProtectedAreas* (19% overall), which had significantly lower results in Zambia (5%). Third in preference was *AntiLogging* (16% effectiveness overall*)*, with no statistically significant differences among countries. The three remaining policy instrument categories had overall effectiveness scores below 10%. Among these, *FinancialTools* showed significantly higher results in Ecuador (25%), *OtherPolicies* had significantly higher effectiveness in Zambia (19%) and *LandUseRights* showed no statistically significant differences between countries.

Concerning the expected effectiveness of policy instrument categories across scales, we could only detect a significantly higher preference for *OtherPolicies* in the regional (subnational) level (14%), when compared to the national (2%) and local levels (6%) (Fig. 4 and Table 3).

### Principal component analysis (PCA)

**Figure 5:**
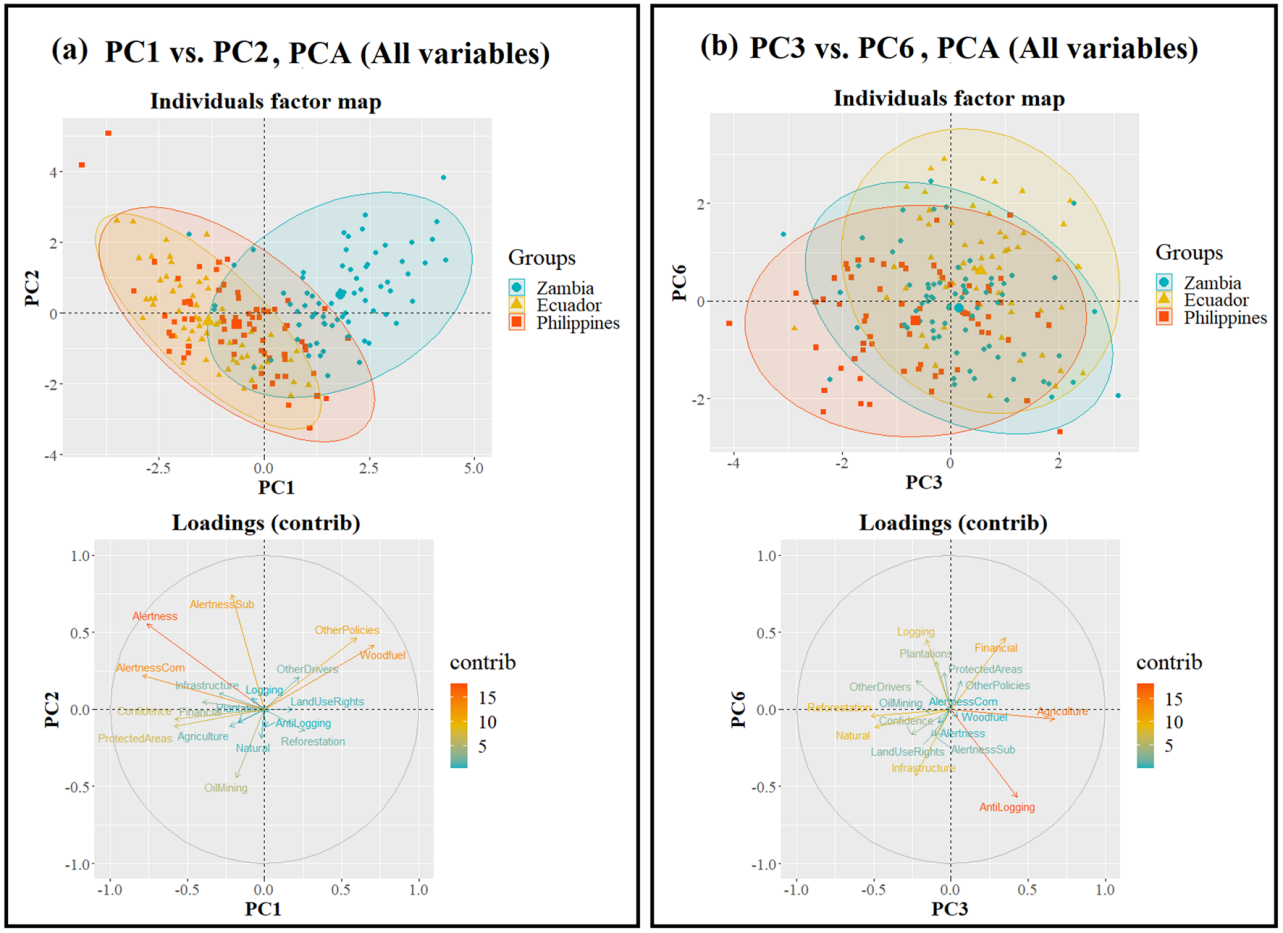
Results of the PCA with all the eighteen studied variables: biplots of the individuals grouped by country (ellipse of 95% confidence) and loadings of the variables for the two first components (**a**) and for the third and sixth component (**b**).

When conducting PCA with all the eighteen studied variables (Supplementary Figs. S9–S11), the first principal component (PC) explained 18.4% of the variance, twice as much as the second PC. The first nine PCs had eigenvalues higher than 1 (Kaiser rule) and explained similar variances ranging from 9.2 (second PC) to 5.9% (eighth PC). Twelve PCs were needed to explain a cumulative variance over 90%. The first PC (Fig. 5) was (strongly) negatively influenced by *Alertness* (overall and for commercial drivers), *Confidence*, *ProtectedAreas* and *FinancialTools*, and positively by *Woodfuel* and *OtherPolicies*. The scores for a number of PCs revealed strong cross-country differences. Specifically, the first two PCs distinguished Zambia from the other two countries, while the third and sixth PCs accounted for variations between Ecuador and the Philippines (Fig. 5). The cross-scale differences were less distinct but still noticeable, especially when observing the scores of the two first PCs (Supplementary Fig. S7).

## Discussion

Our findings indicate that stakeholders in Zambia and those from institutions of subnational levels tend to be less alert about the number of possible commercial threats to forests and are skeptical about the effectiveness of more policy instruments (Table 3 and Fig. 1). However, stakeholders agree across scales about the most important drivers (i.e. agriculture) and about the most effective policy instruments (i.e. reforestation) in the coming decade, which follow regional trends (Table 3 and Figs. 3, 4).

Consistent with our hypothesis, the ANOVA results revealed that *Alertness* and *Confidence* differ across countries (Table 3 and Fig. 1). The PCA ratified the relevance of these two indicators, as they both contributed strongly to the first PC explaining most of the variance and clearly differentiating the Zambian observations (Fig. 5). The differences observed for *Alertness* were attributable to drivers related to the demands of commercial economy. These perceptions align with the regional trends of the last decades, where commercial operators (e.g. agriculture, logging) have been playing a major role in South America and South East Asia, when compared to Africa^17,24^. The fact that Ecuador and especially the Philippines are in a more advanced stage of deforestation^20,43^, could have conditioned the perceptions of their stakeholders to be more alert about potential threats, resulting in a higher perceived need for policy instruments. This is remarkable, as historical deforestation, if related to inefficient policies, can be rather expected as a reason for lower *Confidence*. Ecuador has lost a large share of its native forests since the sixties, catalyzed by agrarian reforms and laws incentivizing land-use conversion and by road construction for the oil industry^54,55^. Similarly, forest cover in the Philippines has decreased drastically from approximately 70% to less than 25% during the twentieth century, mostly due to massive commercial timber harvesting, leading to a nationwide logging moratorium, net wood imports and numerous reforestation and forest restoration programs^56–58^. In contrast, a large share of Zambian primary forests have been degraded since the seventies, but the relatively high forest cover of the country has been decreasing at slower rates^59^. This probably explains the lower *Alertness* in Zambia, while the lower *Confidence* could be rather related to a lack of trust in governance mechanisms, which was recurrently mentioned by the respondents and has been identified by previous research in the region^47,60^. These results may characterize contexts of pre- or early forest transition, when forest areas are still abundant and slow deforestation rates accelerate^17^. This may be seen as a warning sign and point to a need for precautionary measures such as environmental education or the improvement of governance structures^60^, before reaching lower levels of forest cover in Zambia or in countries of the region with similar characteristics (e.g. Gabon, Angola, Tanzania, Liberia, Congo, Democratic Republic of the Congo)^1^. Our results in Ecuador and the Philippines indicate that opposing views are possible, in which the importance of drivers and potential solutions are more strongly taken into consideration by all actors. However, it remains unclear whether this change in perspective can be achieved before assuming uncontrolled deforestation rates and low levels of forest cover in later forest transition stages.

Similarly, lower *Alertness* and *Confidence* detected in institutions of subnational levels (Table 3 and Fig. 1) suggests that international and national stakeholders, normally involved in and responsible for planning and policy design (de jure), would have a broader overview of possible threats and protection mechanisms. Therefore, they would identify a larger set of drivers and policies as having a strong or very strong effect when compared to sub-national and local stakeholders. In contrast, the latter would typically experience a lower number of specific drivers, while being closer to the sometimes-ineffective policy measures being implemented on the ground (de facto)^48,49,60^. Such challenges are especially common in tropical countries characterized by political instability and weak institutions^61^, where the information and rules about political instruments and forest management often reach the local levels with a time delay^62^. Avoiding potential disengagement of local stakeholders regarding national forest protection goals is particularly relevant, as those actors are closer to the effects of deforestation on the ground and closer to reverse such trends with direct action^29,47^. This points to the importance of law enforcement and ensuring economic, logistical and institutional support to local organizations for achieving effective policy implementation^63^. We also interpret that the lower *Alertness* of local stakeholders is related to the fact that they do not perceive deforestation necessarily as a threat, but rather as a potential source of revenue and economic development. Forest products can represent a significant share of total rural household income in the tropics, averaging roughly from 15 to 50% in the studied countries^56,64,65^. This could also be viewed as an explanation for the lower *Alertness* in Zambia, where forest-related share of income was the highest among the target countries^66^. Thus, our results suggest that forest policies or strategies to combat deforestation in the tropics should consider the direct dependence of local, usually rural, populations on forests, to avoid further challenges during implementation.

As hypothesized and supported by the PCA (Fig. 5), the ANOVAs detected significant differences among the three countries regarding the expected importance of the driver categories (Table 3, Figs. 2 and 3). Our findings confirm the higher importance of agricultural expansion and cattle ranching in South America in general^17,24^ and in Ecuador in particular, where they have been responsible for approximately 95% of the forest loss between 1990 and 2018^54^. Similarly, Ecuadorian respondents expected a significantly higher importance for mining and oil extractions. In addition to the historical link between oil development and deforestation^54,55^, recent governmental concessions for such purpose overlap with about 24% of all official indigenous territories and protected areas in the Amazon basin^67^. In Zambia, the higher importance of woodfuel and charcoal production was anticipated in a country where these sources comprise over 70% of the national energy consumption, as they are seen as cheap, accessible and reliable alternatives to electricity^68^. Respondents in Zambia provided a larger number of additional answers, mentioning governance issues as threats to forests in line with previously reported results^47,60^. Zambian stakeholders also reported a significantly lower importance of timber extraction (mostly selective logging leading to degradation^59^) when compared to Ecuador and the Philippines. These two countries present a longer history of combating illegal logging and allowing or prohibiting timber extraction in both private and public lands^69–71^. The responses in the Philippines were relatively high for a larger variety of driver categories, which can also be explained by their history of a nationwide and large-scale deforestation over the twentieth century^56,58,72^. Apart from the mentioned drivers (i.e. *Agriculture*, *Logging* and *OilMining*), the Philippine respondents also highlighted the role of other known threats to forests^72,73^, namely natural disasters (i.e. typhoons, landslides and floods) and infrastructure expansion, the latter without being statistically significant. Thus, our findings indicate that stakeholders expect the currently-relevant drivers to remain important in the future decade, pointing to the continuation of the well-known regional trends and providing hints on which drivers to anticipate and where to do it.

We also observed significant differences among the studied countries regarding the expected effectiveness of policy measures (Table 3, Figs. 2 and 4). In line with current international agenda (e.g. 1 Trillion Trees initiative, Bonn Challenge or the UN Decade on Ecosystem Restoration), reforestation and forest restoration initiatives are the favorite policy instruments overall. The Philippines have reversed the trend of deforestation to a net gain of forest area in the last decade, partly attributed to tree plantings, natural regeneration and high government investments on reforestation projects, such as the National Greening Program^56–58^. The respondents in Ecuador reported a relatively lower effectiveness of *Reforestation* when compared to the Philippines and Zambia, despite several ambitious reforestation plans aiming to convert 300,000 hectares of pastureland to agroforestry systems in the Amazon^74^. This is due to a larger preference for financial instruments, linked to positive experiences regarding the national PES scheme of Socio Bosque^75^. Although protected areas are the second favorite policy instrument overall, their expected effectiveness is half that of reforestation. The Zambian respondents showed a much lower preference for protected areas, likely related to a historic ineffectiveness of such regulatory measures in the country^76^. Policies against illegal logging play a relevant intermediate role and land use rights were less preferred, both without significant differences across countries. Finally, the Zambian respondents (especially the regional subsample), highlighted the importance of other policies, i.e. related to improving governance mechanisms and facilitating energy and livelihood alternatives. Previous results when evaluating the national subsample of our dataset, had already pointed to a similar overall picture about the effectiveness of policy instruments in the tropics^77^.

Overall, the astonishingly high scores of *Reforestation* may indicate a paradigm shift from protected areas to a stronger focus on reforestation and integrative approaches. This points to the importance of including reforestation and forest restoration measures in the design, promotion and management of protected areas and other effective area-based conservation measures (OECMs), which are currently promoted by policy, especially related to biodiversity conservation^78,79^. Reforestation can encompass different forms of natural regrowth, passive/active forest restoration or the establishment of agroforestry areas. These approaches can be relevant in the current context of transformative change towards climate-resilient socioecological systems and the proliferation of fragmented and degraded forests^2,80^. However, we should highlight that this strong preference for reforestation over other policy instruments does not necessarily imply that prioritizing such measures always constitutes sound policy^81,82^. This interesting finding could reflect the widely extended narratives of the current international forest agenda promoting reforestation measures, e.g., in the context of the Bonn Challenge. Similarly, the preference of reforestation measures could point to negative experiences regarding other financial or regulatory policies. For instance, despite positive evidence for the effectiveness of protected areas, these have been found to not always avoid clearing within the boundaries (or to increase the risk in neighbor areas), and to highly depend on monitoring and law enforcement^83–85^.

Surprisingly, we observed very few significant cross-scale differences regarding both the expected importance of deforestation drivers and the expected effectiveness of policy instruments (Table 3, Figs. 3 and 4). First, our findings suggest that subnational stakeholders are more aware of the importance of subsistence activities, such as firewood collection, while national institutions identify commercial and industrial threats of higher importance, such as oil and mining operations. This finding is an example of how scale affects the perception of telecoupled commodities or agricultural trade flows in a globalized economy^86^. Apparently, high-level stakeholders are more concerned about the impacts of such commodities on forests than local actors. Nevertheless, local actors are often both producers and consumers of such commercial products as well. Second, most of the respondents providing suggestions for other policy instruments belonged to the regional level, due to the more detailed recommendations of respondents in academia.

However, the general lack of effects across scales indicates that most of the forest representatives follow the same narratives regarding the main future threats to tropical forests and the favored strategies to combat them, independently of the geographical jurisdiction of their institutions. This contradicts our hypothesis that different stakeholder configurations and interests would result in particular preferences. Concerning policy instruments, for instance, we had expected a favoritism of decentralization measures (i.e. *LandUseRights*) and positive financial incentives (i.e. *FinancialTools*) at local levels, together with a rejection of command-and-control measures such as *ProtectedAreas*, based on our previous work at landscapes of the studied countries^47,60,63^. A possible explanation for this result is the strong influence of the national narratives of success (e.g. Sociobosque in Ecuador) or failure (e.g. protected areas in Zambia) for specific policy instruments, which showed similar degrees of acceptance across spatial levels. This idea would be supported by the fact that most of the interviewed institutions have strong interactions with each other within the national setting, rather than internationally. However, this fact alone would not explain if such dominating and broadly accepted discourses are created unidirectionally and thus dictated by the one or the other stakeholder group (e.g. top-down or bottom-up approaches); or, in contrast, if they are rather the result of a bi-directional exchange of complementary storylines. To achieve these type of conclusions, more complex analysis including the role of power (both over institutions and forest resources) would be needed. In any case, this finding indicates a potential of agreements for future collaboration between actors at different spatial levels, in particular needed for effective policy design and cross-scale implementation of forest conservation measures and forest landscape restoration^24^.

The interpretations and implications of our findings must nonetheless be taken cautiously, as the reliability of this data is impacted by the sample and methodological choices. For instance, the distribution of observations across spatial levels is not perfectly balanced (e.g. less international institutions, more regional stakeholders in Zambia). Also relevant is how the institutions were selected and distributed across spatial levels. The majority of the interviewed stakeholders were representatives (typically men over 45 years of age with university education) of formal government-related institutions. Additionally, the way the answers of importance and effectiveness were collected as compositional data, or the analysis of Likert answers as Top 2 Box scores, conditions the potential for interpretation^87,88^. Another important point is the simplification of categories conducted to generalize our results and make them comparable across countries. In reality, such categories might present strong overlaps or interactions. For instance, drivers of deforestation often act in a conjoint manner, which includes subtle interrelations and dependencies^20^. Similarly, most environmental programs and policies nowadays include a mix or combination of instruments^21,22^, which makes it challenging to assign them or their effects to a specific category. However, the fact that most of PCs explained a similar share of the variance, had eigenvalues close to or higher than 1 and were mostly loaded with one or few variables (Supplementary Fig. S9–S11), indicates that the dimensions could not be reduced easily and that most of the PCs were relevant and related to the included variables. This suggests the independence of the chosen driver and policy categories based on existing literature, confirming their appropriateness in describing distinct deforestation processes and recommending further studies to use similar classifications. Further studies could expand this sample to other tropical countries or extend the representativity of certain stakeholder types. Likewise, we see additional potential in analyzing institutional characteristics such as power, exploring the direct relationships between drivers and policies, or linking perceptions with spatially-explicit data on deforestation for different countries or administrative units.

While the role of the drivers of tropical deforestation and forest degradation in reshaping the Earth’s surface is by now common knowledge, policy instruments often fail to address these drivers effectively across countries and scales. The evidence is clear: local stakeholders and also actors in certain contexts (i.e. Zambia and potentially other African countries with high forest cover) are less alert about a larger number of future commercial threats to tropical forests. In addition, these stakeholders are more skeptical about the effectiveness of existing policy instruments. At the same time, our investigation clearly shows that the national context matters for the perception of both deforestation threats and effective policies, suggesting that there is no one-size-fits-all solution to improve forest policy at a global scale. Despite these differences, actors across scales agree about the most important drivers (i.e. agriculture) and about the most effective policy instruments (i.e. reforestation) in the coming decade. This unexpected consensus confirms the existence of common entry points for collaboration between institutions operating at different spatial levels, which is a precondition for effective policy design and implementation. For instance, the overwhelming favoritism for reforestation and forest restoration initiatives is particularly relevant, as it points to the potential of integrating different forms of reforestation as a complementary component of area-based conservation measures.

## Methods

### Study design

The study was conducted in three tropical countries of Africa (Zambia), South America (Ecuador) and South East Asia (Philippines), as part of the project Landscape Forestry in the Tropics (LaForeT: www.la-foret.org). The country selection aimed to include different continents and a gradient of forest transitions contexts, from early in Zambia (with still a relatively high forest cover and accelerating deforestation rates), middle in Ecuador and late in the Philippines (with historical deforestation resulting in low forest cover and recent reforestation efforts)^43^.

Between November 2018 and December 2019, a total of 224 representatives of forest-related institutions (key informants or stakeholders) were interviewed following a standardized questionnaire. The study sample included respondents from local and central governments, national and international organizations, private enterprises, indigenous associations and academia. An extended list of characteristics of the respondents and their institutions can be found as Supplementary Table S1 online. Additionally, a list of the institutions taking part on the survey, as well as anonymized detailed information on the respondents (i.e. gender, position within the institution, …) and on the institutions themselves (number of workers, type of institution, …) can be found attached to this manuscript as a Supplementary File online (spreadsheet ‘Data Questionnaire’, sheets ‘Institutions’ and ‘Respondents’).

Each of the participants was assigned to one of four spatial levels (Table 1) depending on the nature or main scope of work of the stakeholder’s institution. These spatial levels were related to the different levels of geographical jurisdictions or administrative units located across the spatial scale, i.e. (i) international (e.g. Food and Agriculture Organization or development agencies), (ii) national (e.g. central ministry units or national forestry/environmental departments), (iii) regional (e.g. Provincial offices or sub-national departments/Universities) and (iv) local (e.g. municipal government/offices or traditional leaders). Thus, the regional level captures all subnational jurisdictional units larger or equal to Districts in Zambia, Counties in Ecuador and Provinces in the Philippines. The local level comprises institutions with a scope at smaller jurisdictional units (e.g. Chiefdoms in Zambia, Parishes in Ecuador, Municipalities or Barangays in the Philippines).

### Questionnaire

The protocol of the questionnaire study was approved through research permits signed by all participating scientific institutions, namely the Thünen Institute in Hamburg (Germany), the Universidad Estatal Amazónica in Puyo (Ecuador), the University of the Philippines in Los Baños (Philippines) and the Copperbelt University in Kitwe (Zambia). The methods were carried out in accordance with the guidelines of good scientific practice from the German Research Foundation (DFG) and relevant regulations. Informed consent was obtained from all participants, who were all over eighteen years of age at the time of conducting the interviews.

Our questionnaire included two sections: (i) one, asking about the influence of different proximate drivers on deforestation and forest degradation in the next 10 years; and (ii) a second one, asking about the influence of policy measures on stopping deforestation/degradation and increasing forest areas, again in the future 10 years. Based on existing literature and expert knowledge^47,58,63,89–91^, we provided a list of nationally relevant drivers and policy instruments for each section respectively and gave the respondents the opportunity to add their own answers. In the case of the drivers, we focused on proximate or direct drivers, while the policy instruments included regulatory (i.e. spatial planning direct regulation), economic (i.e. land tenure, positive/negative incentives, market mechanisms) and information instruments. We further aggregated all the drivers and policy instruments into eight and six cross-country categories, respectively. These categories were defined based on the literature mentioned in the introduction^8,17,21,22^ and to be broad enough to include a sufficiently high number of answers for comparison across countries and spatial levels. Thus, the aggregated results for cross-country categories included a varying number of answers for multiple national drivers and policy instruments. A detailed list of questions/answers for both sections of the questionnaire in the three countries, grouped by cross-country categories, are included as supplementary information (see Supplementary Tables S2, S3 online).

The respondents could score each national driver or policy instrument based on a Likert scale^92^, from 1 (no effect) to 5 (very strong effect). In the case of the first section, the participants could also distinguish if the drivers were related to the demands of subsistence or commercial economy. Additionally, the respondents listed their top three to five important national drivers and policies, respectively, each with a share of relative relevance adding up to 100. An extended version of these results can be found as Supplementary Figs. S1 to S8. All the answers were collected in digital format and included in a common database for the three countries by the project staff. The complete list of responses for all the drivers and policies with details on national and cross-country categories, including the Likert and rank/percentage answers, can be found attached to this manuscript as a Supplementary File online (spreadsheet ‘Data Questionnaire’, sheet ‘Responses’).

### Variables

Based on the answers of the respondents, we derived a total of eighteen variables per questionnaire (Table 2), which were further used in the statistical analyses. We include descriptive statistics of all these variables for the total sample and for the country and spatial level subsamples as Supplementary Tables S4, S5 online. The complete list of values for all the variables and observation units (respondents) can be found as a Supplementary File online (spreadsheet ‘Data Questionnaire’, sheet ‘Variables’).

#### Overall alertness about deforestation drivers and overall confidence in policy instruments

From the Likert answers we derived two variables: (i) “Overall alertness about deforestation drivers” (*Alertness*) and (ii) “Overall confidence in policy measures” (*Confidence*). These two variables were defined as the share of answers with “strong” (4) or “very strong” (5) influence in each section of the questionnaire, respectively (Top 2 Box scores [T2B] in percentage^93^). We included these variables as indicators of the stakeholders’ general perception about the influence of drivers and policy instruments. As described above, in the case of the driver categories (*Alertness*), we could also further distinguish between the answers related to the demands of subsistence (*AlertnessSub*) or commercial (*AlertnessCom*) economy.

#### Expected importance of deforestation drivers and expected effectiveness of policy instruments

By adding up the answers on relative relevance in percentage, we derived fourteen further variables, related to the expected relative importance and effectiveness of the specific cross-country driver and policy instrument categories, respectively. Thus, we calculated the expected relative importance of the following eight drivers: (i) Expansion of agriculture (*Agriculture*), (ii) Logging, timber and resource extraction (*Logging*), (iii) Firewood, woodfuel and charcoal (*Woodfuel*), (iv) Oil and mining (*OilMining*), (v) Infrastructure and urbanization (*Infrastructure*), (vi) Expansion of timber plantations (*Plantations*), (vii) Natural disasters (*NaturalDisasters*) and (viii) Other drivers (*OtherDrivers*). Correspondingly, we calculated the expected relative effectiveness of the following six policy instruments: (i) Reforestation, restoration and agroforestry (*Reforestation*), (ii) Protected areas (*ProtectedAreas*), (iii) Measures against logging (*AntiLogging*), (iv) Financial instruments (*FinancialTools*), (v) Land-use rights (*LandUseRights*) and (vi) Other policy instruments (*OtherPolicies*).

### Statistical analysis

For all the steps described in this section we used R^94^ packages rstatix^95^ and factoextra^96^, as well as multiple helper functions^97–104^. The complete R script used for the analysis can be found as a Supplementary File, attached to this manuscript online.

First, we checked the distribution of each variable by analyzing visually the histograms and boxplots, before and after centering, scaling and selecting a transformation (square-root, log or inverse), which brought the skewness the closest to 0. We confirmed the visual interpretations by performing Shapiro–Wilk tests^105^ of univariate normality and Mardia^106^ tests of multivariate normality (see Supplementary Tables S6, S7).

We could not find significant evidence of multivariate or univariate normality for most of the selected variables. This was expected due to the type of survey data used (i.e. Likert scores and compositional data), known for presenting particular properties (e.g. presence of zeroes, not enough observations for particular answers, ordinal scales) which result in mathematical challenges when applying parametric methods^87,88^.

In addition, we removed questionnaires with errors, missing entries or outliers before each of the specific statistical analysis. From the original 224 interviews, this resulted in 218 observations including valid responses about alertness, 217 about confidence and 219 and 203 questionnaires including valid answers about the importance of driver and policy categories, respectively (Supplementary Tables S4, S5).

#### One-way analysis of variance (ANOVA)

We conducted parametric and non-parametric one-way ANOVA for all the studied variables across countries and across spatial scales, to test whether the different samples originated from the same distribution. As we could not confirm normality, we relied on the results of the non-parametric Kruskal–Wallis one-way ANOVA^107^, accompanied by Dunn’s test^108^ and pairwise Mann–Whitney tests with Bonferroni correction^109^ (Supplementary Tables S8, S9). Nevertheless, we also conducted parametric one-way ANOVAs (by generalizing the *t* statistic^110^ to three [country] and four [spatial level] samples) and pairwise Tukey test^111^, in order to compare and support the validity of our results (Supplementary Tables S10, S11).

#### Principal component analysis (PCA)

We conducted PCA^112^ with all the eighteen studied variables (scaled), in order to find relationships and correlations within them and further support the interpretation of the ANOVAs across countries and spatial levels. With this approach, we also aimed to explore if the number of pre-selected categories could be reduced and still capture most of the variation in the answers The extended results for these tests, including spree plots, proportions of variance explained, eigenvalues, loadings and biplots of the first components (PCs) are included as supplementary information (see Supplementary Figs. S9–S11).

## Supplementary Information

Supplementary Information 1.

Supplementary Information 2.

Supplementary Information 3.

## Data Availability

Data supporting the results reported in the manuscript (without breaching participant confidentiality) is freely available to any researcher wishing to use them for non-commercial purposes, in the Supplementary Information files of these article (Spreadsheet file ‘Data Questionnaire’).

## References

[1] Global Forest Resources Assessment 2020 (FAO, 2020).

[2] Taubert F (2018). Global patterns of tropical forest fragmentation. Nature.

[3] Vancutsem C (2021). Long-term (1990–2019) monitoring of forest cover changes in the humid tropics. Sci. Adv..

[4] Foley JA (2007). Amazonia revealed: Forest degradation and loss of ecosystem goods and services in the Amazon Basin. Front. Ecol. Environ..

[5] Barlow J (2018). The future of hyperdiverse tropical ecosystems. Nature.

[6] Brandon K (2014). Ecosystem services from tropical forests: Review of current science. SSRN J..

[7] Indarto, J. & Mutaqin, D. J. An overview of theoretical and empirical studies on deforestation. MPRA. Paper No. 70178 (2016).

[8] Geist HJ, Lambin EF (2002). Proximate causes and underlying driving forces of tropical deforestation: Tropical forests are disappearing as the result of many pressures, both local and regional, acting in various combinations in different geographical locations. Bioscience.

[9] Angelsen A, Kaimowitz D (1999). Rethinking the causes of deforestation: Lessons from economic models. World Bank Res. Obs..

[10] Contreras-Hermosilla A (2000). The Underlying Causes of Forest Decline.

[11] Turner BL (1990). Two types of global environmental change: Definitional and spatial-scale issues in their human dimensions. Glob. Environ. Change.

[12] Meyer WB, Turner BL (1992). Human population growth and global land-use/cover change. Ann. Rev. Ecol. Syst..

[13] Miyamoto M, Mohd Parid M, Noor Aini Z, Michinaka T (2014). Proximate and underlying causes of forest cover change in Peninsular Malaysia. For. Policy Econ..

[14] Lim CL, Prescott GW, De Alban JDT, Ziegler AD, Webb EL (2017). Untangling the proximate causes and underlying drivers of deforestation and forest degradation in Myanmar. Conserv. Biol..

[15] Carodenuto S (2015). A methodological framework for assessing agents, proximate drivers and underlying causes of deforestation: Field test results from southern cameroon. Forests.

[16] Curtis PG, Slay CM, Harris NL, Tyukavina A, Hansen MC (2018). Classifying drivers of global forest loss. Science.

[17] Hosonuma N (2012). An assessment of deforestation and forest degradation drivers in developing countries. Environ. Res. Lett..

[18] Köthke M, Leischner B, Elsasser P (2013). Uniform global deforestation patterns—An empirical analysis. For. Policy Econ..

[19] Busch J, Ferretti-Gallon K (2017). What drives deforestation and what stops it? A meta-analysis. Rev. Environ. Econ. Policy.

[20] Ferrer Velasco RF, Köthke M, Lippe M, Günter S (2020). Scale and context dependency of deforestation drivers: Insights from spatial econometrics in the tropics. PLoS One.

[21] Lambin EF (2014). Effectiveness and synergies of policy instruments for land use governance in tropical regions. Glob. Environ. Change.

[22] Börner J, Schulz D, Wunder S, Pfaff A (2020). The effectiveness of forest conservation policies and programs. Ann. Rev. Resour. Econ..

[23] Bemelmans-Videc M-L, Rist RC, Vedung E (1998). Carrots, Sticks & Sermons: Policy Instruments and their Evaluation.

[24] Seymour F, Harris NL (2019). Reducing tropical deforestation. Science.

[25] Lambin EF (2018). The role of supply-chain initiatives in reducing deforestation. Nat. Clim. Change.

[26] Wolff S, Schweinle J (2022). Effectiveness and economic viability of forest certification: A systematic review. Forests.

[27] Müller R, Pistorius T, Rohde S, Gerold G, Pacheco P (2013). Policy options to reduce deforestation based on a systematic analysis of drivers and agents in lowland Bolivia. Land Use Policy.

[28] Tegegne YT, Lindner M, Fobissie K, Kanninen M (2016). Evolution of drivers of deforestation and forest degradation in the Congo Basin forests: Exploring possible policy options to address forest loss. Land Use Policy.

[29] Hoffmann C, García Márquez JR, Krueger T (2018). A local perspective on drivers and measures to slow deforestation in the Andean-Amazonian foothills of Colombia. Land Use Policy.

[30] Henders S, Ostwald M, Verendel V, Ibisch P (2018). Do national strategies under the UN biodiversity and climate conventions address agricultural commodity consumption as deforestation driver?. Land Use Policy.

[31] Salvini G (2014). How countries link REDD+ interventions to drivers in their readiness plans: implications for monitoring systems. Environ. Res. Lett..

[32] Bos AB (2020). Integrated assessment of deforestation drivers and their alignment with subnational climate change mitigation efforts. Environ. Sci. Policy.

[33] Fritz S (2022). A continental assessment of the drivers of tropical deforestation with a focus on protected areas. Front. Conserv. Sci..

[34] Lawrence D, Vandecar K (2015). Effects of tropical deforestation on climate and agriculture. Nat. Clim. Change.

[35] Fedele G, Locatelli B, Djoudi H, Colloff MJ (2018). Reducing risks by transforming landscapes: Cross-scale effects of land-use changes on ecosystem services. PLoS One.

[36] Yackulic CB (2011). Biophysical and socioeconomic factors associated with forest transitions at multiple spatial and temporal scales. Ecol. Soc..

[37] Loran C, Ginzler C, Bürgi M (2016). Evaluating forest transition based on a multi-scale approach: Forest area dynamics in Switzerland 1850–2000. Reg. Environ. Change.

[38] Moonen PC (2016). Actor-based identification of deforestation drivers paves the road to effective REDD+in DR Congo. Land Use Policy.

[39] Strassburg, B. The tragedy of the tropics: A dynamic, cross-scale analysis of deforestation incentives. *Working Paper—Centre for Social and Economic Research on the Global Environment* No. 07-02 (2007).

[40] López-Carr D (2012). Space versus place in complex human–natural systems: Spatial and multi-level models of tropical land use and cover change (LUCC) in Guatemala. Ecol. Model..

[41] Hoang NT, Kanemoto K (2021). Mapping the deforestation footprint of nations reveals growing threat to tropical forests. Nat. Ecol. Evol..

[42] Pendrill F (2019). Agricultural and forestry trade drives large share of tropical deforestation emissions. Glob. Environ. Change.

[43] Ferrer Velasco R (2022). Towards accurate mapping of forest in tropical landscapes: A comparison of datasets on how forest transition matters. Remote Sens. Environ..

[44] Jayathilake HM, Prescott GW, Carrasco LR, Rao M, Symes WS (2021). Drivers of deforestation and degradation for 28 tropical conservation landscapes. Ambio.

[45] Minang PA (2014). REDD+Readiness progress across countries: Time for reconsideration. Clim. Policy.

[46] Current pledges | Bonn challenge. https://www.bonnchallenge.org/pledges. Accessed: 15th August 2022.

[47] Nansikombi H (2020). Can de facto governance influence deforestation drivers in the Zambian Miombo?. For. Policy Econ..

[48] Sullivan A, York A, White D, Hall S, Yabiku SD (2017). Jure versus de facto institutions: Trust, information, and collective efforts to manage the invasive mile-a-minute weed (*Mikania micrantha*). Int. J. Commons.

[49] Busch J, Amarjargal O (2020). Authority of second-tier governments to reduce deforestation in 30 tropical countries. Front. For. Glob. Change.

[50] Sandström C, Eckerberg K, Raitio K (2013). Studying conflicts, proposing solutions—Towards multi-level approaches to the analyses of forest conflicts. For. Policy Econ..

[51] Hoogstra-Klein MA, Permadi DB, Yasmi Y (2012). The value of cultural theory for participatory processes in natural resource management. For. Policy Econ..

[52] de Jong W, Ruiz S, Becker M (2006). Conflicts and communal forest management in northern Bolivia. For. Policy Econ..

[53] Eckerberg K, Sandström C (2013). Forest conflicts: A growing research field. For. Policy Econ..

[54] Sierra, R., Calva, O. & Guevara, A. *La Deforestación en el Ecuador, 1990–2018. Factores promotores y tendencias recientes*, 216 (2021).

[55] Wasserstrom R, Southgate D (2013). Deforestation, agrarian reform and oil development in Ecuador, 1964–1994. Nat. Resour..

[56] Wiebe PC, Zhunusova E, Lippe M, Ferrer Velasco R, Günter S (2022). What is the contribution of forest-related income to rural livelihood strategies in the Philippines’ remaining forested landscapes?. For. Policy Econ..

[57] Le HD, Smith C, Herbohn J (2014). What drives the success of reforestation projects in tropical developing countries? The case of the Philippines. Glob. Environ. Change.

[58] Carandang, A. P. et al. Analysis of key drivers of deforestation and forest degradation in the Philippines. *Deutsche Gesellschaft für Internationale Zusammenarbeit (GIZ)* (2013).

[59] Phiri D, Morgenroth J, Xu C (2019). Four decades of land cover and forest connectivity study in Zambia—An object-based image analysis approach. Int. J. Appl. Earth Obs. Geoinf..

[60] Nansikombi H, Fischer R, Kabwe G, Günter S (2020). Exploring patterns of forest governance quality: Insights from forest frontier communities in Zambia’s Miombo ecoregion. Land Use Policy.

[61] Zhang H, Wang P, Wood J, Wood J, Chaiechi T, Thirumaran K (2022). Does institutional quality matter for the nexus between environmental quality and economic growth?: A tropics perspective. Business, Industry, and Trade in the Tropics.

[62] Reed J, Van Vianen J, Deakin EL, Barlow J, Sunderland T (2016). Integrated landscape approaches to managing social and environmental issues in the tropics: Learning from the past to guide the future. Glob. Change Biol..

[63] Fischer R (2021). Interplay of governance elements and their effects on deforestation in tropical landscapes: Quantitative insights from Ecuador. World Dev..

[64] Torres B, Vasco C, Günter S, Knoke T (2018). Determinants of agricultural diversification in a hotspot area: Evidence from colonist and indigenous communities in the Sumaco biosphere reserve Ecuadorian Amazon. Sustainability.

[65] Ojeda Luna T, Zhunusova E, Günter S, Dieter M (2020). Measuring forest and agricultural income in the Ecuadorian lowland rainforest frontiers: Do deforestation and conservation strategies matter?. For. Policy Econ..

[66] Kazungu M (2021). Effects of household-level attributes and agricultural land-use on deforestation patterns along a forest transition gradient in the Miombo landscapes Zambia. Ecol. Econ..

[67] Kleemann J (2022). Deforestation in continental ecuador with a focus on protected areas. Land.

[68] Mulenga MM, Roos A (2021). Assessing the awareness and adoptability of pellet cookstoves for low-income households in Lusaka, Zambia. J. Energy South. Afr..

[69] Eguiguren P, Ojeda Luna T, Torres B, Lippe M, Günter S (2020). Ecosystem service multifunctionality: Decline and recovery pathways in the amazon and chocó lowland rainforests. Sustainability.

[70] Vasco C, Torres B, Pacheco P, Griess V (2017). The socioeconomic determinants of legal and illegal smallholder logging: Evidence from the Ecuadorian Amazon. For. Policy Econ..

[71] van der Ploeg J, van Weerd M, Masipiqueña AB, Persoon GA (2011). Illegal logging in the Northern Sierra Madre Natural Park, the Philippines. Conserv. Soc..

[72] Liu DS, Iverson LR, Brown S (1993). Rates and patterns of deforestation in the Philippines: Application of geographic information system analysis. For. Ecol. Manag..

[73] Boquet Y, Boquet Y (2017). Environmental challenges in the Philippines. The Philippine Archipelago.

[74] MAGAP. *ATPA: Reconversión Agro productiva Sostenible en la Amazonía Ecuatoriana* (2014).

[75] Jones KW (2017). Forest conservation incentives and deforestation in the Ecuadorian Amazon. Environ. Conserv..

[76] Lindsey PA (2014). Underperformance of African protected area networks and the case for new conservation models: Insights from Zambia. PLoS One.

[77] Fischer R (2022). Effectiveness of policy instrument mixes for forest conservation in the tropics – a stakeholder perspective from Ecuador, the Philippines and Zambia. Land Use Policy.

[78] Gurney GG (2021). Biodiversity needs every tool in the box: Use OECMs. Nature.

[79] Maxwell SL (2020). Area-based conservation in the twenty-first century. Nature.

[80] Priebe J (2022). Transformative change in context—Stakeholders’ understandings of leverage at the forest–climate nexus. Sustain. Sci..

[81] Höhl M (2020). Forest landscape restoration—What generates failure and success?. Forests.

[82] Köthke M, Ahimbisibwe V, Lippe M (2022). The evidence base on the environmental, economic and social outcomes of agroforestry is patchy—An evidence review map. Front. Environ. Sci..

[83] Fischer R, Giessen L, Günter S (2020). Governance effects on deforestation in the tropics: A review of the evidence. Environ. Sci. Policy.

[84] Bare M, Kauffman C, Miller DC (2015). Assessing the impact of international conservation aid on deforestation in sub-Saharan Africa. Environ. Res. Lett..

[85] Vuohelainen AJ, Coad L, Marthews TR, Malhi Y, Killeen TJ (2012). The effectiveness of contrasting protected areas in preventing deforestation in Madre de Dios. Peru. Environ. Manag..

[86] Hull V, Liu J (2018). Telecoupling: A new frontier for global sustainability. Ecol. Soc..

[87] Aitchison J (1982). The statistical analysis of compositional data. J. Roy. Stat. Soc..

[88] Norman G (2010). Likert scales, levels of measurement and the “laws” of statistics. Adv. Health Sci. Educ..

[89] Day M, Gumbo D, Moombe KB, Wijaya A, Sunderland T (2014). Zambia Country Profile: Monitoring, Reporting and Verification for REDD+.

[90] Piotrowski, M. Nearing the tipping point. Drivers of Deforestation in the Amazon Region (2019).

[91] Sarker PK, Fischer R, Tamayo F, Navarrete BT, Günter S (2022). Analyzing forest policy mixes based on the coherence of policies and the consistency of legislative policy instruments: A case study from Ecuador. For. Policy Econ..

[92] Likert R (1932). A technique for the measurement of attitudes. Arch. Psychol..

[93] Altinsoy M (2021). Ambulatory ECG monitoring for syncope and collapse in United States, Europe, and Japan: The patients’ viewpoint. J. Arrhythm..

[94] R Core Team. *R: A language and environment for statistical computing*. https://www.R-project.org/. (R Foundation for Statistical Computing, Vienna, Austria, 2022).

[95] Kassambara, A. rstatix: Pipe-friendly framework for basic statistical tests. R package version 0.7.0 (2021).

[96] Kassambara, A. & Mundt, F. factoextra: Extract and visualize the results of multivariate data analyses. R package version 1.0.7 (2020).

[97] Komsta, L. & Novometsky, F. moments: Moments, cumulants, skewness, kurtosis and related tests. R package version 0.14.1 (2022).

[98] Zhang, Y., Zhou, M. & Shao, Y. mvnormalTest: Powerful tests for multivariate normality. R package version 1.0.0 (2020).

[99] Kassambara, A. ggpubr: ‘ggplot2’ Based Publication Ready Plots. R package version 0.4.0 (2020).

[100] Wickham H (2019). Welcome to the Tidyverse. JOSS.

[101] Bache, S. M. & Wickham, H. magrittr: A Forward-Pipe Operator for R. R package version 2.0.3 (2022).

[102] Ushey, K., Allaire, J., Wickham, H. & Ritchie, G. *rstudioapi: Safely Access the RStudio API. R package version 0.13* (2020).

[103] Wickham H (2016). ggplot2: Elegant Graphics for Data Analysis.

[104] Wilkins, D. treemapify: Draw Treemaps in ‘ggplot2’. R package version 2.5.5 (2021).

[105] Shapiro SS, Wilk MB (1965). An analysis of variance test for normality (complete samples). Biometrika.

[106] Mardia KV (1970). Measures of multivariate skewness and kurtosis with applications. Biometrika.

[107] Kruskal WH, Wallis WA (1952). Use of ranks in one-criterion variance analysis. J. Am. Stat. Assoc..

[108] Dunn OJ (1964). Multiple comparisons using rank sums. Technometrics.

[109] Conover WJ, Iman RL (1981). Rank transformations as a bridge between parametric and nonparametric statistics. Am. Stat..

[110] Student (1908). The probable error of a mean. Biometrika.

[111] Tukey JW (1949). Comparing individual means in the analysis of variance. Biometrics.

[112] Jolliffe IT (2002). Principal Component Analysis.

