# Supplementary material for: Reconciling policy instruments with drivers of deforestation and forest degradation: cross-scale analysis of stakeholder perceptions in tropical countries
Source: Sci Rep. 2023 Feb 7;13:2180. doi: 10.1038/s41598-023-29417-y (PMC9905477; doi:10.1038/s41598-023-29417-y)
Supplement: Supplementary file 1 — Supplementary Information 1. [file 41598_2023_29417_MOESM1_ESM.pdf]

Supplementary Information

Reconciling policy instruments with drivers of deforestation and forest degradation: Cross-scale analysis of stakeholder perceptions in tropical countries

Rubén Ferrer Velasco[1,2*], Melvin Lippe[2], Richard Fischer[2], Bolier Torres[3], Fabián Tamayo[3], Felix Kanungwe Kalaba[4], Humphrey Kaoma[4], Leonida Bugayong[5], and Sven Günter[1,2]

[1] Ecosystem Dynamics and Forest Management Group, School of Life Sciences, Technical University of Munich (TUM), 85354 Freising, Germany
[2] Institute of Forestry, Johann Heinrich von Thünen Institute, 21031 Hamburg, Germany
[3] Life Sciences Department, Universidad Estatal Amazónica (UEA), 160101 Puyo, Ecuador
[4] School of Natural Resources, Copperbelt University, 21692 Kitwe, Zambia
[5] Forestry Development Center, College of Forestry and Natural Resources, University of the Philippines Los Baños, 4031 Laguna, Philippines
[*] ruben.weber@tum.de

Supplementary Information

Ferrer Velasco et al. Reconciling policy instruments with drivers of deforestation and forest degradation: Cross-scale analysis of stakeholder perceptions in tropical countries

Table of contents

1. Descriptive statistics of stakeholders and institutions 1

Supplementary Table S1. Number of interviews conducted per country (ZMB: Zambia, ECU: Ecuador, PHL: Philippines) and spatial level of institution (INT: International, NAT: National, REG: Regional, LOC: Local), grouped by characteristics of the respondents and by the characteristics of the stakeholders' institutions. 1

2. Lists of driver and policy instrument answers/categories 3

Supplementary Table S2. Pre-selection of answers (and extra answers provided by the respondents) to the questions: "Please score for each of the following drivers their influence on deforestation and forest degradation in the next 10 years" and "Which of the presented deforestation and forest degradation drivers will be the TOP 3 to 5 in the next 10 years?", in each country and grouped by cross-country driver categories. 3

Supplementary Table S3. Pre-selection of answers (and extra answers provided by the respondents) to the questions: "Please score for each of the following Reforestation and Conservation measures their influence on reforestation or forest conservation in the next 10 years?" and "Which of the Reforestation and Conservation measures will be the TOP 3 to 5 measures that can stop deforestation and increase forest areas in the next 10 years?", in each country and grouped by cross-country policy instrument categories (second section of the questionnaire). 4

3. Answers about drivers of deforestation and forest degradation (Likert and top-5 rankings). 6

Supplementary Figure S1. Total number (N) and percentage distribution of Likert responses regarding the influence of drivers on deforestation and forest degradation in the next 10 years due to the demands of commercial (C) / subsistence (S), for the total sample (a) and country subsamples (b, c, d). Blank answers are not shown or considered for the total N in the graph. The bars are plotted so that the bar corresponding to the center of the Likert scale is centered at 0. 6

Supplementary Figure S2. Total number (N) and percentage distribution of Likert responses regarding the influence of drivers on deforestation and forest degradation in the next 10 years due to the demands of commercial (C) / subsistence (S), for the spatial level subsamples (a, b, c, d). Blank answers are not shown or considered for the total N in the graph. The bars are plotted so that the bar corresponding to the center of the Likert scale is centered at 0. 7

Supplementary Figure S3. Number of responses ranked within the top-5 per driver category, regarding the influence on deforestation and forest degradation in the next 10 years, for the total sample (a) and the country subsamples (b, c, d). Note: the range of the x-axis varies between samples. Blank answers are not shown or considered for the total N in the graph. 8

Supplementary Figure S4. Number of responses ranked within the top-5 per driver category, regarding the influence on deforestation and forest degradation in the next 10 years, for the four spatial level subsamples. Note: the range of the x-axis varies between samples. Blank answers are not shown or considered for the total N in the graph. 9

4. Answers about policy instruments (Likert and top-5 rankings). 10

Supplementary Figure S5. Total number (N) and percentage distribution of Likert responses regarding the influence of policy instruments on forest protection in the next 10 years, for the total sample (a) and the national subsamples (b, c, d). Blank answers are not shown or considered for the total N in the graph. 10

Supplementary Figure S6. Total number (N) and percentage distribution of Likert responses regarding the influence of policy instruments on forest protection in the next 10 years, for the spatial level subsamples (a, b, c, d). Blank answers are not shown or considered for the total N in the graph 11

Supplementary Figure S7. Number of responses ranked within the top-5 per policy instrument category, regarding the influence on forest protection in the next 10 years, for the total sample (a) and the country subsamples (b, c, d). Note: the range of the x-axis varies between samples. Blank answers are not shown or considered for the total N in the graph. 12

Supplementary Figure S8. Number of responses ranked within the top-5 per policy instrument category, regarding the influence on forest protection in the next 10 years, for the four spatial level subsamples. Note: the range of the x-axis varies between samples. Blank answers are not shown or considered for the total N in the graph. 13

5. Summary statistics for al variables across countries and across spatial scales 14

Supplementary Table S4. Summary statistics (count, average and standard deviation) of the studied variables for the total sample and the country subsamples. 14

Supplementary Table S5. Summary statistics (count, average and standard deviation) of the studied variables for the spatial level subsamples. 15

6. Variables and transformations: analyzing univariate and multivariate normality 16

Supplementary Table S6. Skewness, histograms, boxplots, Shapiro-Wilk and Mardia normality results for the variables related to drivers of deforestation, before and after transformation (we selected the method which brought skewness the closest to zero, between square-root, log or inverse). 16

Supplementary Table S7. Skewness, histograms, boxplots, Shapiro-Wilk and Mardia normality results for the variables related to policy instruments, before and after transformation (we selected the method which brought skewness the closest to zero, between square-root, log or inverse). 18

7. Principal component analysis (PCA) 20

Supplementary Figure S9. Results of the PCA with all the variables: spree plot (a) showing the percentage of explained variances of the ten first principal components, table (b) listing the standard deviation (sdev), eigenvalue (eigenv), variance (var) and cumulative variance (cumvar) of each principal component and table (c) listing the average score of each principal component, grouped by country and spatial level 20

Supplementary Figure S10. Results of the PCA with all the variables: correlation plot (a) and table (b) showing the loadings of the different principal components by policy instrument category. (…continues…) 21

Supplementary Figure S11. Results of the PCA with all the variables: biplots of the individuals grouped by country (a) and spatial level (b) and of the variables (driver categories) (c) for the two first components. 23

8. Non-parametric analysis of variance: one-way Kruskal-Wallis and Dunn tests 24

Supplementary Table S8. Results of the Kruskal-Wallis and Dunn tests for all driver-related variables across countries and spatial levels, including significance (sign, ****: <0.0001, ***: <0.001, **: <0.01, *: <0.05, ns: not significant [>0.05]) total and cross-groups (Zmb: Zambia, Ecu: Ecuador, Phl: Philippines Int: International, Nat: National, Reg: Regional, Loc: Local), degrees of freedom (df), chi square statistic (χ2), effect size partial eta squared (effsize) and magnitude (magn, lar: large, mod: moderate, sma: small). See Figure S22. 24

Supplementary Table S9. Results of the Kruskal-Wallis and Dunn tests for all the policy-related variables across countries and spatial levels, including significance (sign, ****: <0.0001, ***: <0.001, **: <0.01, *: <0.05, ns: not significant [>0.05]) total and cross-groups (Zmb: Zambia, Ecu: Ecuador, Phl: Philippines, Int: International, Nat: National, Reg: Regional, Loc: Local), chi square statistic (χ2), effect size partial eta squared (effsize) and magnitude (magn, lar: large, mod: moderate, sma: small). See Figure S22. 25

9. Parametric analysis of variance: one-way ANOVA and Tukey multiple comparison of means 26

Supplementary Table S10. Results of the parametric one-way ANOVA and Tukey tests for all driver-related variables across countries and spatial levels, including significance (sign, ****: <0.0001, ***: <0.001, **: <0.01, *: <0.05, ns: not significant [>0.05]) overall and cross-groups (Zmb: Zambia, Ecu: Ecuador, Phl: Philippines, Int: International, Nat: National, Reg: Regional, Loc: Local), degrees of freedom (df), sum and mean of squares (SumSq, MeanSq), F-Values, mean differences (diff.) and confidence intervals (lower, upper). (…continues…) 26

Supplementary Table S11. Results of the parametric one-way ANOVA and Tukey tests for all policy-related variables across countries and spatial levels, including significance (sign, ****: <0.0001, ***: <0.001, **: <0.01, *: <0.05, ns: not significant [>0.05]) overall and cross-groups (Zmb: Zambia, Ecu: Ecuador, Phl: Philippines, Int: International, Nat: National, Reg: Regional, Loc: Local), degrees of freedom (df), sum and mean of squares (SumSq, MeanSq), F-Values, mean differences (diff.) and confidence intervals (lower, upper). (…continues…) 29

References 31

1. Descriptive statistics of stakeholders and institutions

Supplementary Table S1. Number of interviews conducted per country (ZMB: Zambia, ECU: Ecuador, PHL: Philippines) and spatial level of institution (INT: International, NAT: National, REG: Regional, LOC: Local), grouped by characteristics of the respondents and by the characteristics of the stakeholders' institutions.

		Country			Spatial level of institution				
**Characteristics of respondents**		*ZMB*	*ECU*	*PHL*	*INT*	*NAT*	*REG*	*LOC*	**Total**
**Country**	*Zambia*	-	-	-	6	16	35	16	**73**
	Ecuador	-	-	-	7	28	18	13	**66**
	Philippines	-	-	-	5	38	19	23	**85**
**Gender**	*Female*	9	14	25	1	29	11	7	**48**
	Male	55	52	60	16	53	57	41	**167**
	Unknown	9	0	0	1	0	4	4	**9**
**Age**	*Under 30*	0	0	4	1	3	0	0	**4**
	30 - 44	0	0	13	0	7	2	4	**13**
	45 - 59	0	0	52	3	19	13	17	**52**
	More than 60	0	0	11	1	4	4	2	**11**
	Unknown	73	66	5	13	49	53	29	**144**
**Education**	*No University*	0	0	4	0	0	2	2	**4**
	Undergraduate	0	0	47	3	22	6	16	**47**
	Postgraduate (Master)	0	0	20	1	14	2	3	**20**
	Postgraduate (PhD)	0	0	11	1	1	8	1	**11**
	Unknown	73	66	3	13	45	54	30	**142**
**Characteristics of the institutions**									
**Size of institution**	*(1-5)*	12	4	2	1	5	8	4	**18**
**(N workers)**	*(6-10)*	12	19	3	2	15	10	7	**34**
	(11-50)	17	17	17	9	20	15	7	**51**
	(51-200)	17	18	26	3	19	25	14	**61**
	(>200)	6	8	20	2	17	10	5	**34**
	No answer	9	0	17	1	6	4	15	**26**

…continues…

… continuation Table S1 …

		Country			Spatial level of institution				
		ZMB	*ECU*	*PHL*	*INT*	*NAT*	*REG*	*LOC*	**Total**
**Type of institution**	*Central government*	40	24	46	1	60	41	8	**110**
	Local government	0	15	9	0	0	5	19	**24**
	Indigenous associations	8	4	9	0	0	7	14	**21**
	National environmental NGOs	5	5	9	0	17	1	1	**19**
	Private enterprises	10	6	3	3	4	2	10	**19**
	Academia & Research	5	6	6	0	1	16	0	**17**
	International organizations	5	6	3	14	0	0	0	**14**
**Resources[1]**	*Very insufficient, it impedes work*	10	3	19	1	4	18	9	**32**
	Insufficient	29	14	53	2	40	37	17	**96**
	Enough to perform most tasks, but still gaps	2	29	10	5	18	10	8	**41**
	Sufficient for main tasks	13	12	1	2	12	3	9	**26**
	Enough to fulfill all tasks timely and in good quality	10	8	0	8	7	2	1	**18**
	No answer	9	0	2	0	1	2	8	**11**
**Total**		**73**	**66**	**85**	**18**	**82**	**72**	**52**	**224**

[1]: Answer of the respondents to the question: "*My organization has adequate resources (staff with up-to-date knowledge and skills, technology and equipment, budget) to perform its tasks in the field of SFM, de-/reforestation.*"

Note: More than three fourths of the respondents (77%) were male representatives. We only obtained information about age and education in the Philippines, where the majority of the interviewed stakeholders (75%) were older than 45 years of age and possessed a university degree (95%), generally an undergraduate one (57%). Our sample was largely dominated by institutions related to central governments (50% of the conducted questionnaires). Most of these central institutions belonged to the national or the regional spatial levels (60 and 41 out of 110, respectively). The other six stakeholder groups studied (e.g., academia, private enterprises, indigenous associations) represented similar smaller shares of the total sample, ranging from 6% (i.e., international organizations) to 10% (i.e., local governments). Approximately half of the interviewed representatives belonged to large organizations with more than 50 workers. Moreover, eight out of ten respondents declared gaps or insufficient resources for their forest institutions to perform their tasks. This perception of not having adequate resources was especially recurrent in the Philippines, with 96% of the participants acknowledging gaps.

2. Lists of driver and policy instrument answers/categories

Supplementary Table S2. Pre-selection of answers (and extra answers provided by the respondents) to the questions: "Please score for each of the following drivers their influence on deforestation and forest degradation in the next 10 years" and "Which of the presented deforestation and forest degradation drivers will be the TOP 3 to 5 in the next 10 years?", in each country and grouped by cross-country driver categories.

**Cross-country driver categories**[5–9]	**Country**[1–4]		
	**Zambia**	**Ecuador**	**Philippines**
**Expansion of agriculture (*Agriculture*)**	*Agriculture*<br>*Expansion of agroforestry systems*<br>*Expansion of pasture areas*<br>*Shifting cultivation*	*Expansion of annual crops*<br>*Expansion of permanent crops*<br>*Expansion of agroforestry*<br>*Expansion of pasture areas*	*Land-use conversion for agriculture*<br>*Expansion of kaingin (locally common practice of slush-and-burn farming)*<br>*Expansion of highland*
**Logging, timber and resource extraction (*Logging*)**	*Selected logging/timber extraction*<br>*Tree felling licenses*<br>*Forest concession licenses*<br>*Extraction for construction materials*<br>*Illegal activities/harvesting*<br>*Caterpillar collection by felling trees*	*Selected logging/timber extraction*<br>*Tree harvest licenses from PAFSI/PAFSU*<br>*Extraction for construction materials*<br>*Illegal activities*	*Legal and illegal resource extraction*<br>*Timber poaching*<br>*Tree cutting for rattan extraction*<br>*Tree cutting for agar wood*
**Firewood, woodfuel and charcoal (*Woodfuel*)**	*Wood fuel*<br>*Firewood*<br>*Charcoal*	*Firewood and charcoal production*	*Firewood and charcoal production*
**Oil and mining (*OilMining*)**	*Mining activities*	*Oil/mining activities*	*Tree cutting for small-scale mining*<br>*Mining activities (exploration)*
**(*Infrastructure*) and Urbanization**	*Infrastructure development*	*Infrastructure development*<br>*Roads (driven by wood industry)*	*Settlement*<br>*Infrastructure*
**Expansion of timber (*Plantations*)**	*Expansion of timber plantations*	*Expansion of timber plantations*	*Expansion of plantation*
**Natural disasters (*Natural*)**	*Natural disasters: drought fires flooding etc.*	*Natural disasters: drought fires flooding etc.*	*Natural disasters: drought fires floods landslides earthquakes etc.*
**Other drivers (*OtherDrivers***	*Foreign trade*<br>*Low education, lack of knowledge*<br>*Political interference*	*Invasions, "colonos"*<br>*Illegal appropriation of states' land*<br>*Lack of watershed management*	-

Supplementary Table S3. Pre-selection of answers (and extra answers provided by the respondents) to the questions: "Please score for each of the following Reforestation and Conservation measures their influence on reforestation or forest conservation in the next 10 years?" and "Which of the Reforestation and Conservation measures will be the TOP 3 to 5 measures that can stop deforestation and increase forest areas in the next 10 years?", in each country and grouped by cross-country policy instrument categories (second section of the questionnaire).

Cross-country policy instrument categories[11,12]	Country[2,4,10]		
	**Zambia**	**Ecuador**	**Philippines**
**Reforestation restoration, agroforestry (*Reforestation*)**	*Establishment of community woodlots*<br>*Establishment of Agroforestry areas*<br>*Reforestation*<br>*Regrowth of natural forest successions*<br>*Mining practices restoration measures*	*Reforestation with commercial aims*<br>*Reforestation with conservation aims*<br>*Regrowth of natural forest successions*<br>*Enrichment*<br>*Reforestation with SFM*	*Reforestation programs in forestlands*<br>*National Greening Program (NGP)*<br>*ACIAR (Restoration)*<br>*Rainforestation*<br>*Accelerated Pioneer Climax Species (APCS)*<br>*Urban greening*<br>*Indigenous reforestation system (Moyong)*
**Protected areas (*ProtectedAreas*)**	*State forest reserves*<br>*Local forest reserves*<br>*Private forest reserves*	*SNAP*<br>*Bosques Protectores*<br>*Support of indigenous reserves*<br>*Indigenous conservation agreements*	*Protected area management*<br>*Ancestral Domain management*<br>*LAWIN, Mining Forest Program…*<br>*Ref. & Cons. programs in protected areas*
**Measures against logging (AntiLogging)**	*Logging ban in natural forests*<br>*Stronger controls to prevent illegal logging (patrolling, regulate exports, forest rangers, subdistrict monitoring)*	*Introduce a logging ban in natural forests*<br>*Stronger controls to prevent illegal logging (e.g., forest patrolling)*	*EO23 Moratorium logging natural forest*<br>*Stronger controls to prevent illegal logging (MFPC, Anti-illegal TF, Bantay-Gubat, Checkpoints, National Forest Stocks System)*
**Financial instruments (*Financial*)**	*Certification (FSC)*<br>*Business-funded incentives*<br>*PES for successful restoration/SFM*<br>*National policy under REDD+*<br>*Improved financial support*	*Certification (FSC/PEFC)*<br>*Biomprendimientos, Incentives*<br>*Financial support small business*<br>*SocioBosque, REDD+, PES, Corporate Social Responsibility, IFC, RSPO*	*REDD+*
**Land-use rights (*LandUseRights*)**<br><br>**(…continues…)**	*Improved and secured land titling*<br>*Enhance local participation of tradition al leaders in NRM, decentralization,*<br>*Consistent land-use plans*	*Improved and secured land titling (private holders, indigenous comm..)*<br>*Improved census*	*Community-based Forest Management Program (CBFMA, PACBRAMA)*<br>*Integrated Forest Management Program (IFMA, SIFMA)*

(… continuation Table S3…)

Cross-country policy instrument categories	Country: Zambia	Country: Ecuador	Country: Philippines
**Other policy instruments (*OtherPolicies*)**	*Children/youth environmental activities*<br>*Introduce/promote alternative energy sources, Cooking stoves*<br>*Charcoal from pine branches*<br>*Promote alternative livelihood activities (e.g., beekeeping)*<br>*Reduce political interference, Increase coordination, Sensitization, Zoning*<br>*Ban traditional braziers, controlled burning*	*Eliminate negative incentives*<br>*Other international initiatives (FIAS-REE, ITTO, NGOs)*<br>*International support*	*Other international initiatives (AFOCO, JICA, INREMP, FASPO, GIZ, SECAL, ECOGOV, RP, UNDP, FAO)*<br>*CSO initiatives, CSR, Local funding*

3. Answers about drivers of deforestation and forest degradation (Likert and top-5 rankings).

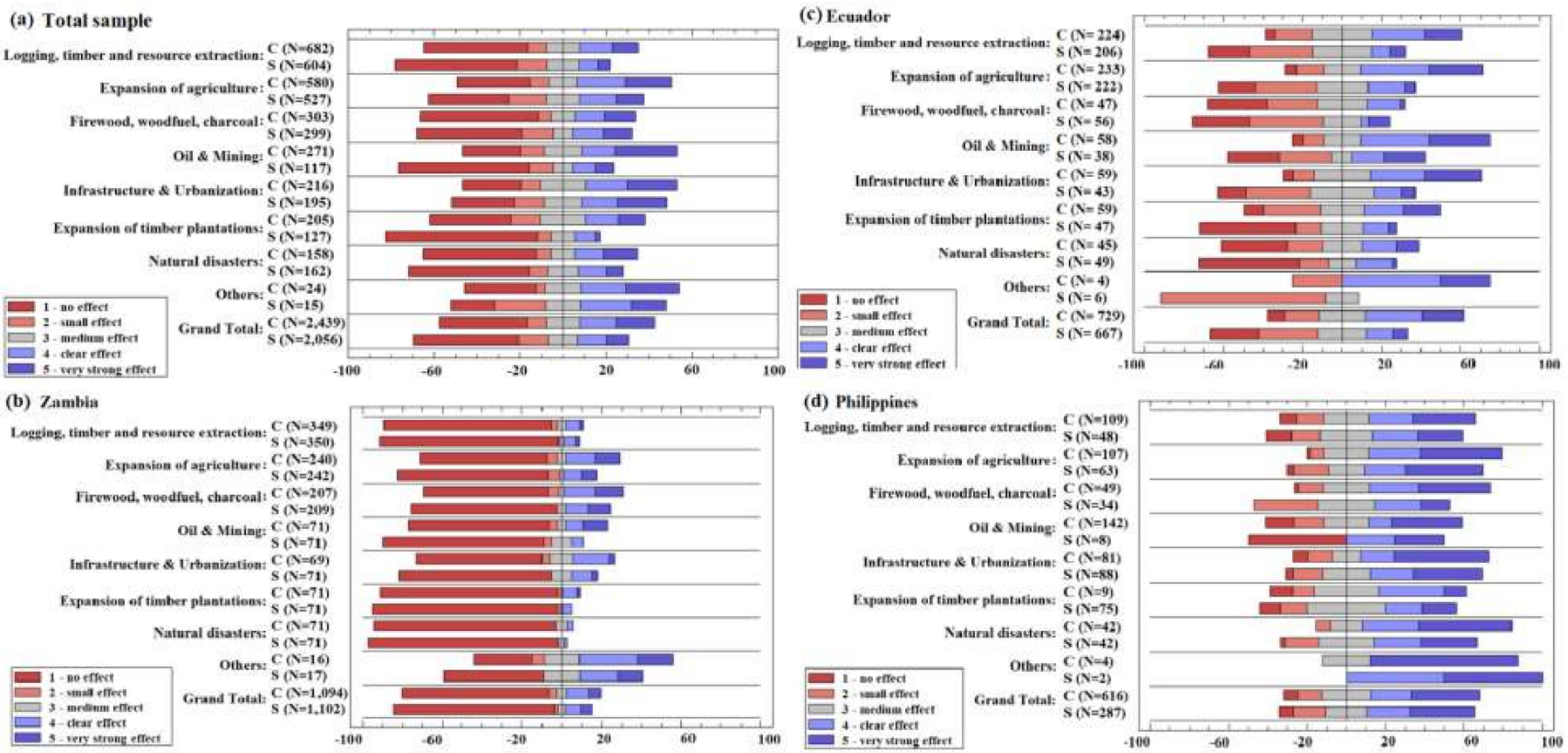

Supplementary Figure S1. Total number (N) and percentage distribution of Likert responses regarding the influence of drivers on deforestation and forest degradation in the next 10 years due to the demands of commercial (C) / subsistence (S), for the total sample (a) and country subsamples (b, c, d). Blank answers are not shown or considered for the total N in the graph. The bars are plotted so that the bar corresponding to the center of the Likert scale is centered at 0.

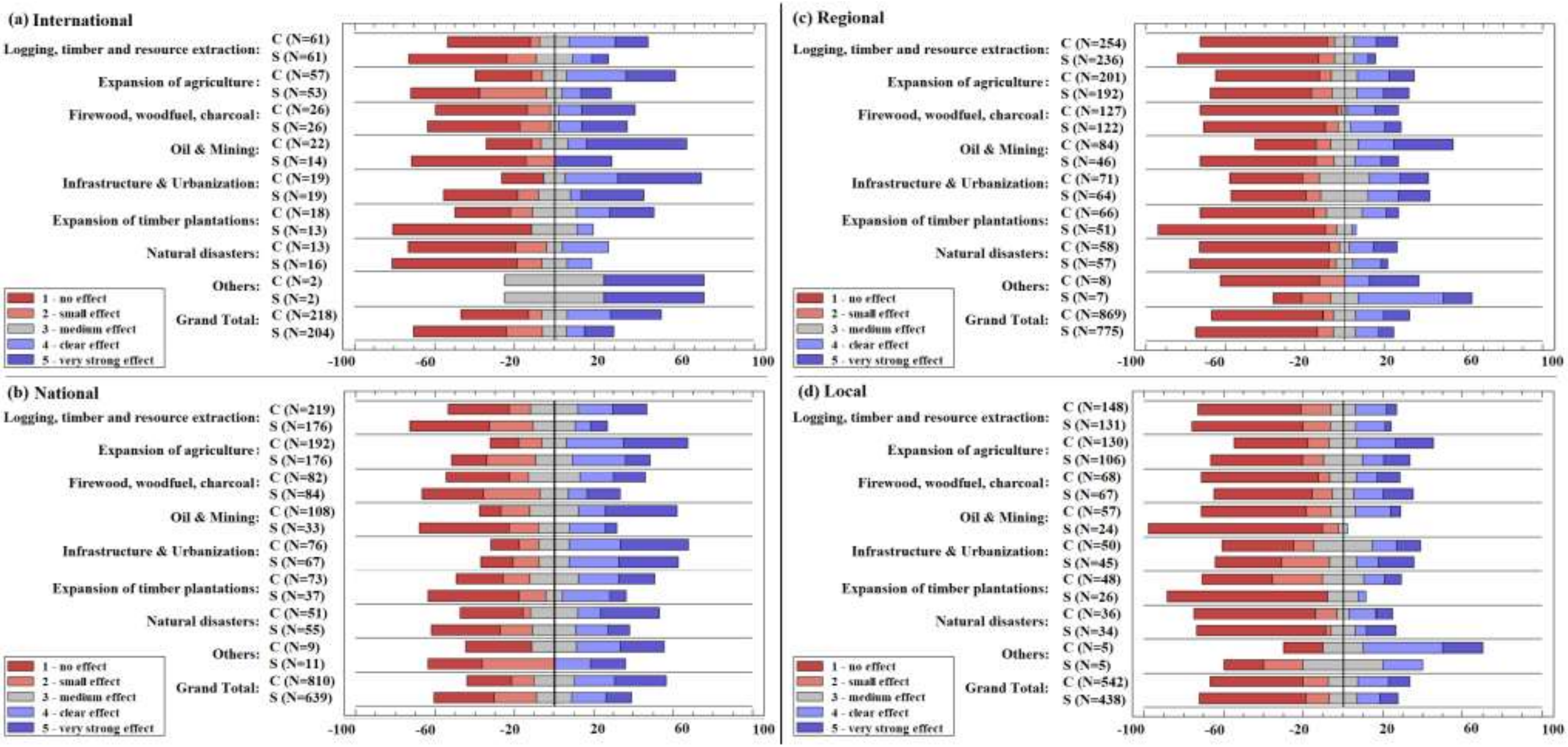

Supplementary Figure S2. Total number (N) and percentage distribution of Likert responses regarding the influence of drivers on deforestation and forest degradation in the next 10 years due to the demands of commercial (C) / subsistence (S), for the spatial level subsamples (a, b, c, d). Blank answers are not shown or considered for the total N in the graph. The bars are plotted so that the bar corresponding to the center of the Likert scale is centered at 0.

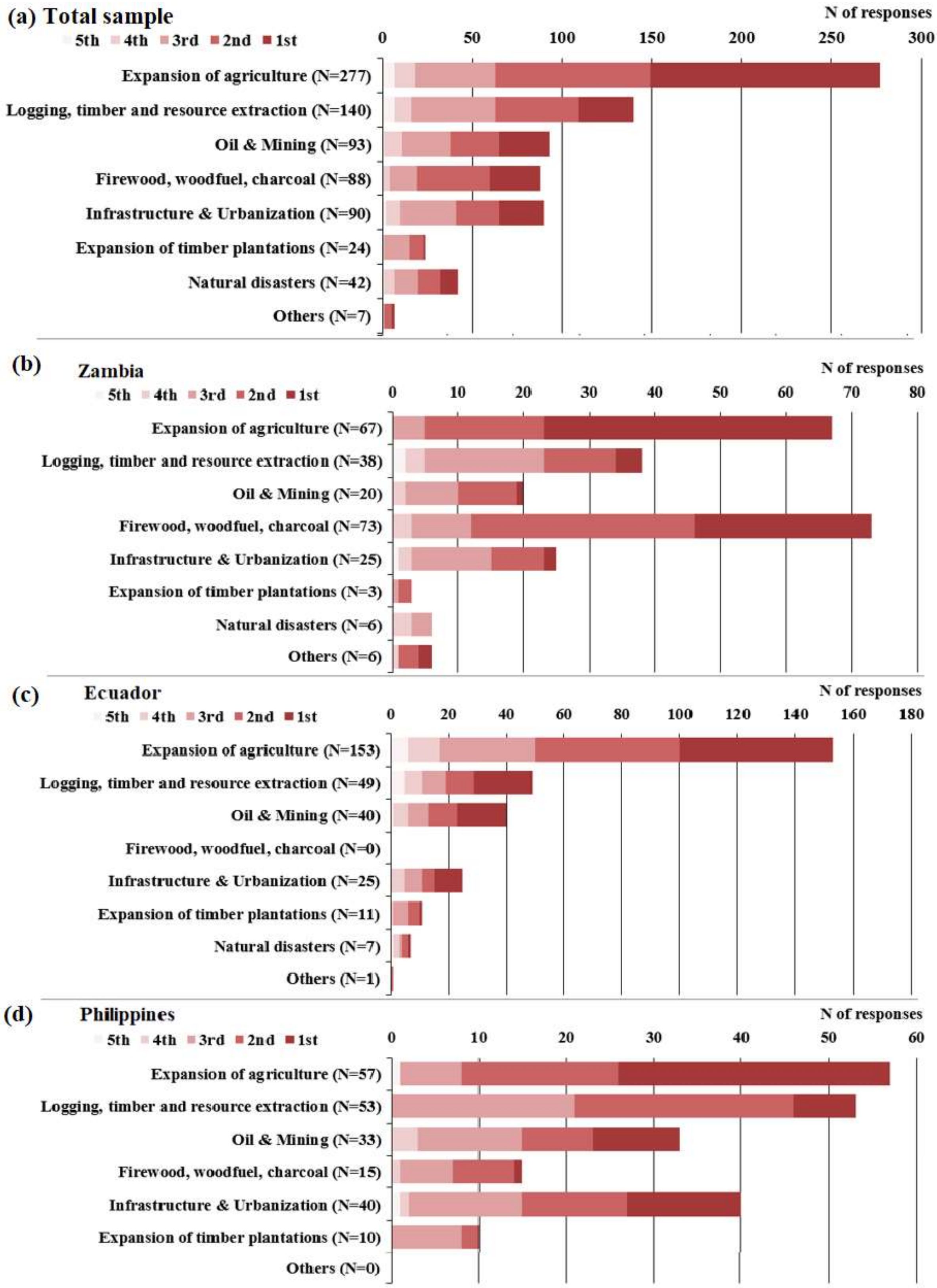

Supplementary Figure S3. Number of responses ranked within the top-5 per driver category, regarding the influence on deforestation and forest degradation in the next 10 years, for the total sample (a) and the country subsamples (b, c, d). Note: the range of the x-axis varies between samples. Blank answers are not shown or considered for the total N in the graph.

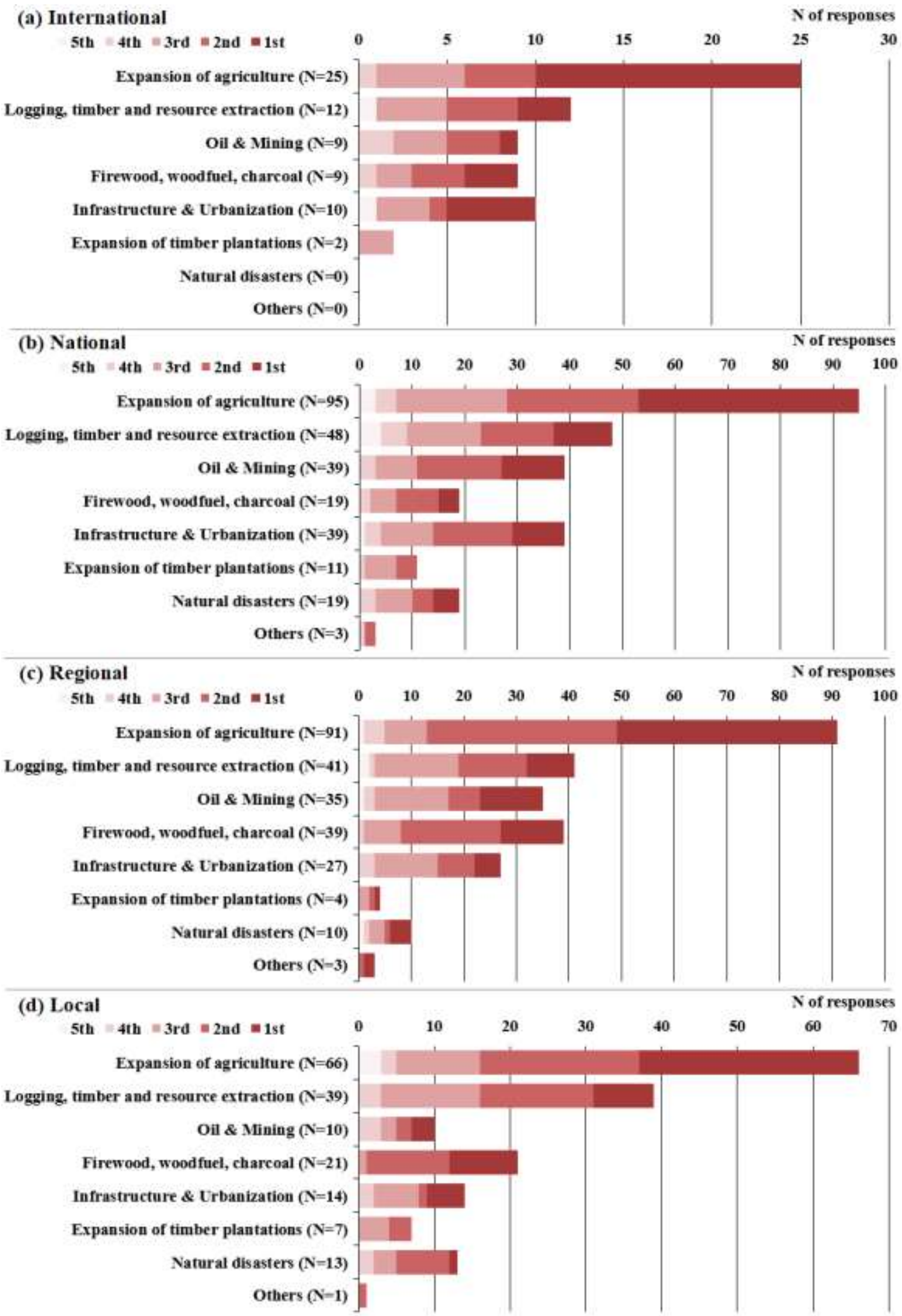

Supplementary Figure S4. Number of responses ranked within the top-5 per driver category, regarding the influence on deforestation and forest degradation in the next 10 years, for the four spatial level subsamples. Note: the range of the x-axis varies between samples. Blank answers are not shown or considered for the total N in the graph.

4. Answers about policy instruments (Likert and top-5 rankings).

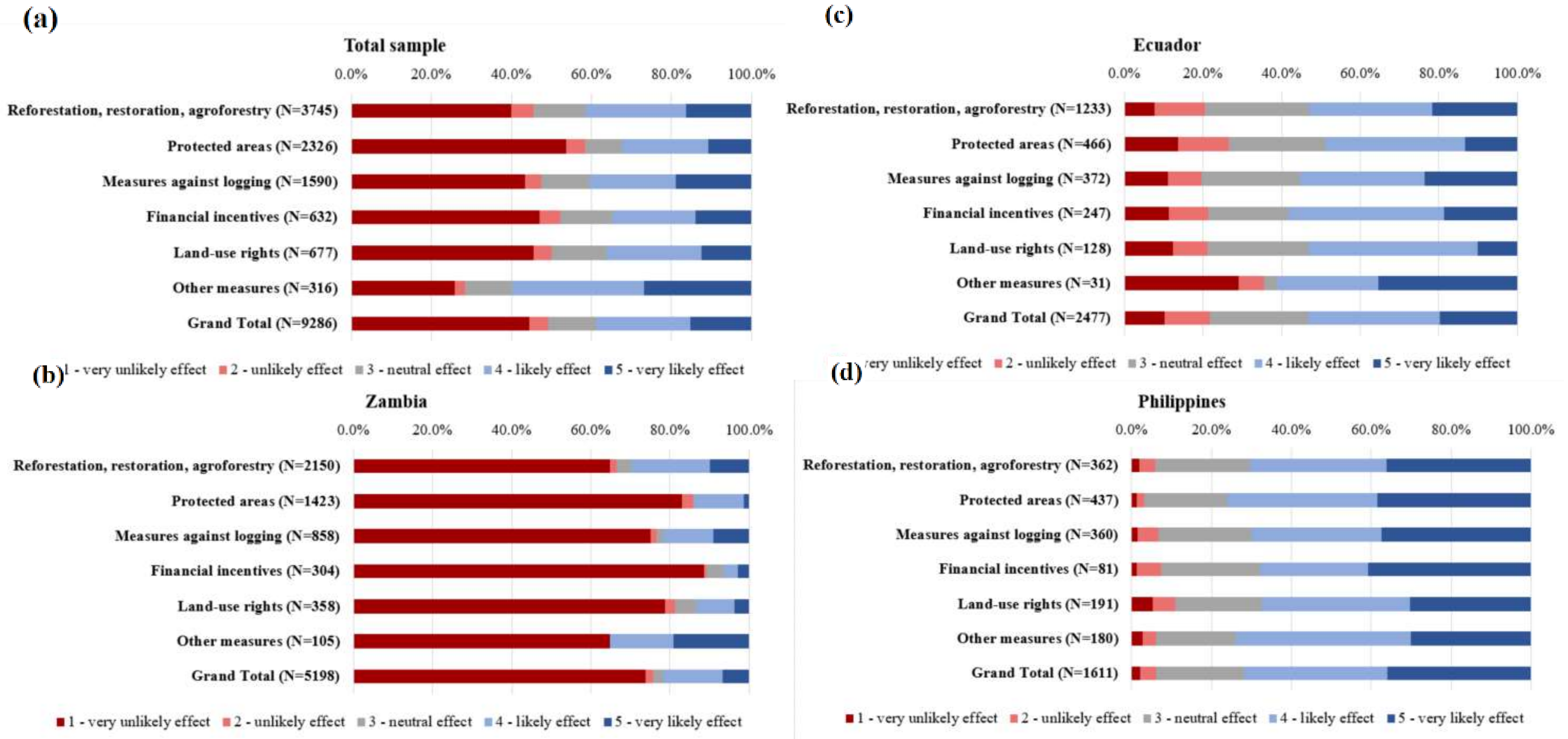

Supplementary Figure S5. Total number (N) and percentage distribution of Likert responses regarding the influence of policy instruments on forest protection in the next 10 years, for the total sample (a) and the national subsamples (b, c, d). Blank answers are not shown or considered for the total N in the graph.

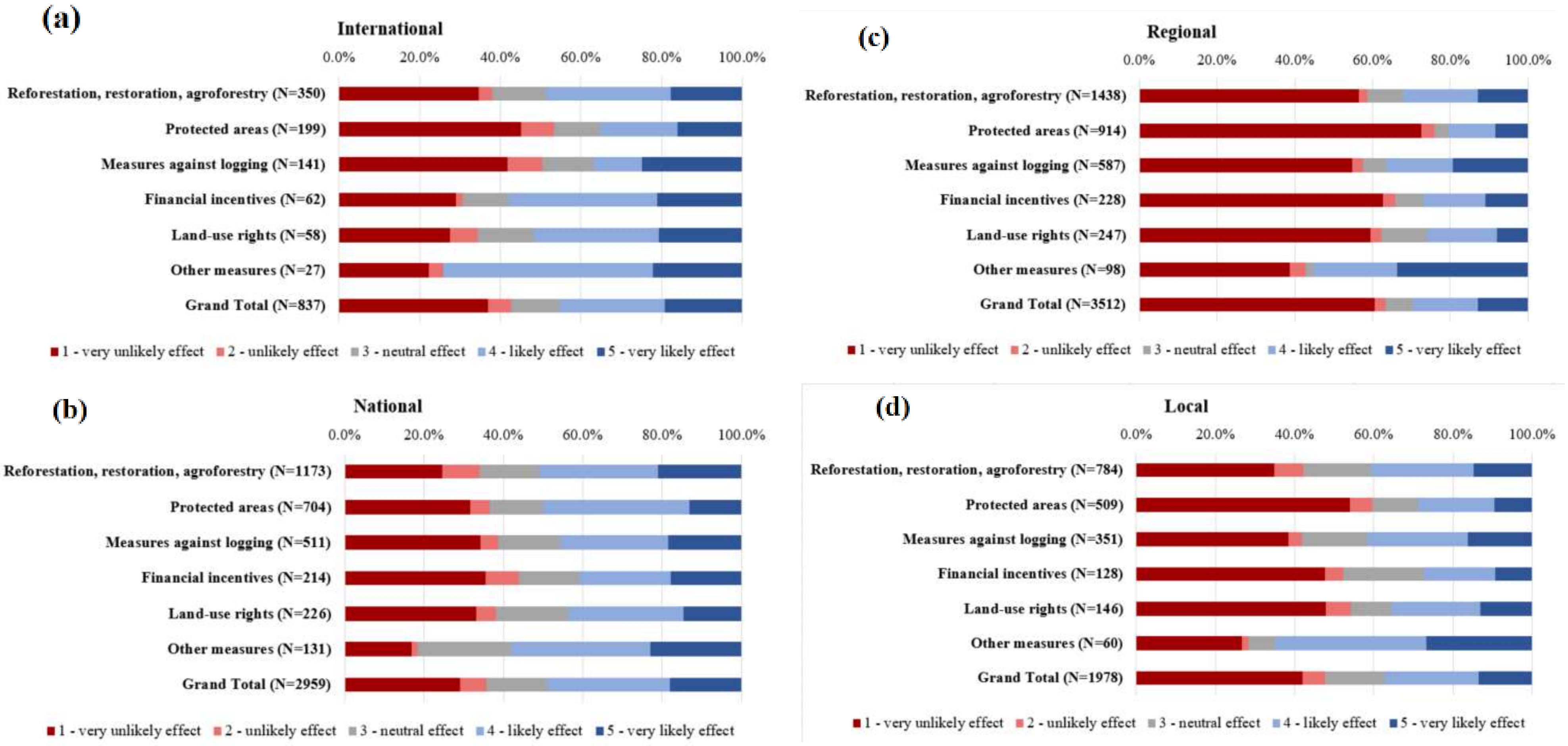

Supplementary Figure S6. Total number (N) and percentage distribution of Likert responses regarding the influence of policy instruments on forest protection in the next 10 years, for the spatial level subsamples (a, b, c, d). Blank answers are not shown or considered for the total N in the graph

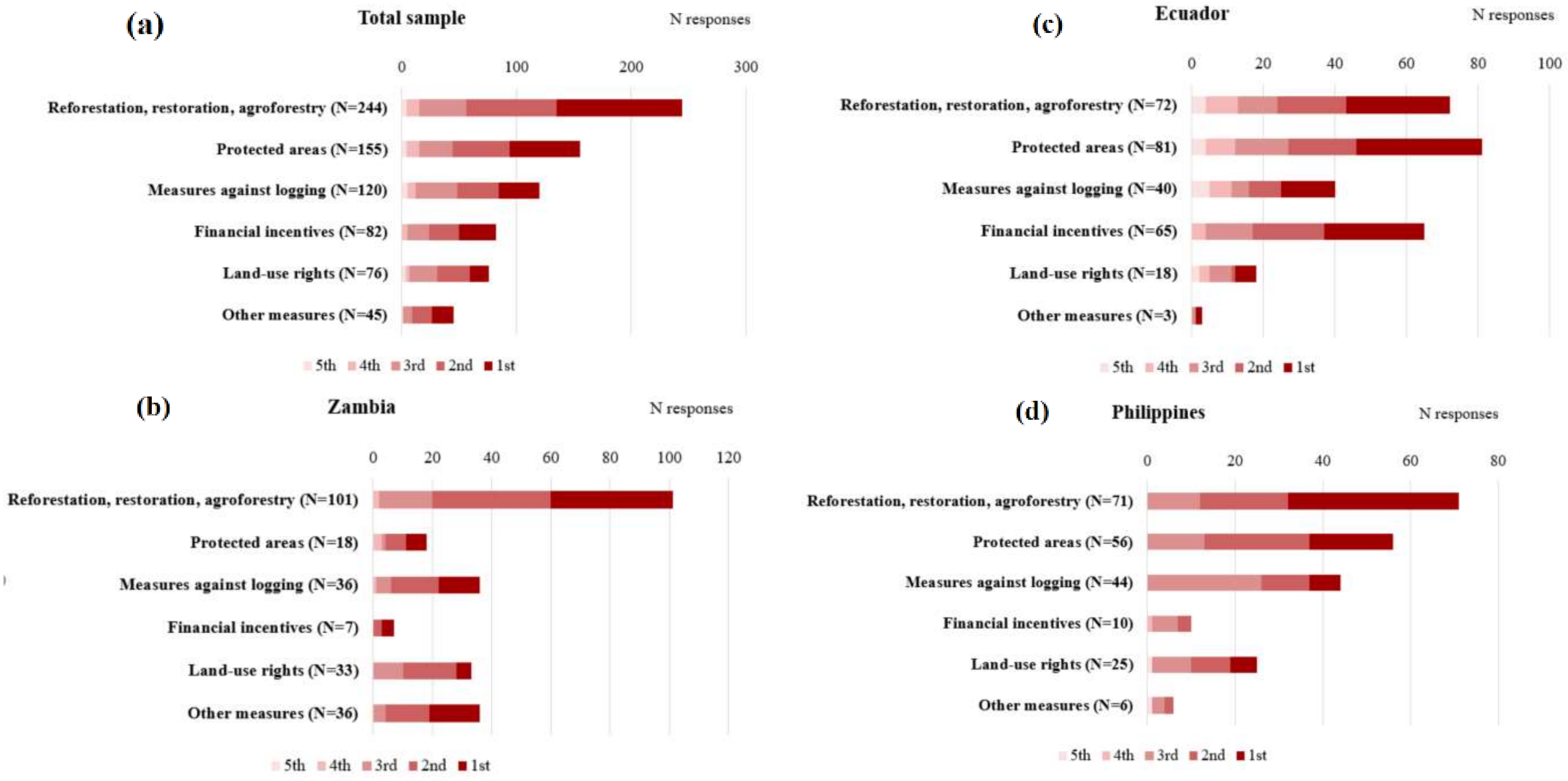

Supplementary Figure S7. Number of responses ranked within the top-5 per policy instrument category, regarding the influence on forest protection in the next 10 years, for the total sample (a) and the country subsamples (b, c, d). Note: the range of the x-axis varies between samples. Blank answers are not shown or considered for the total N in the graph.

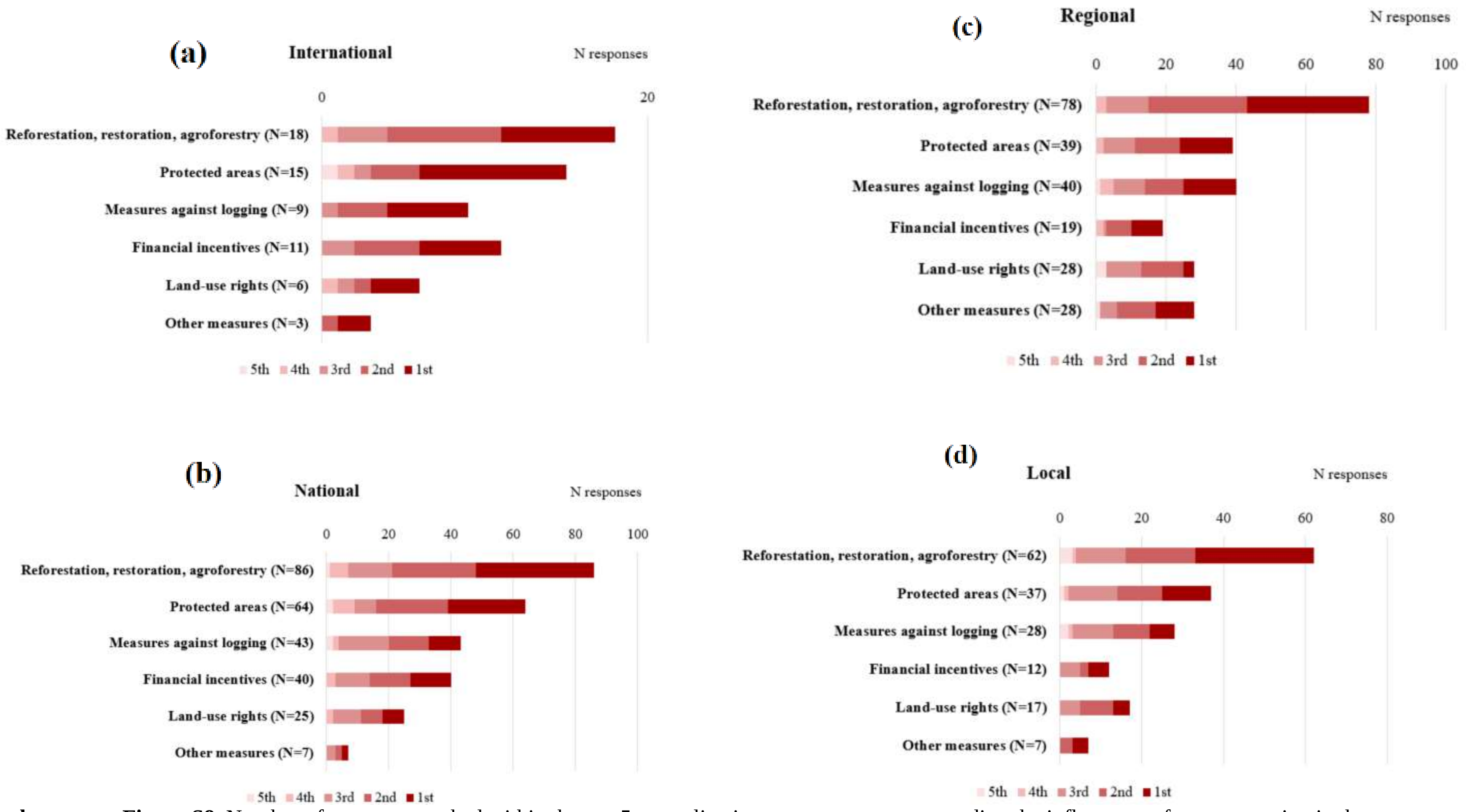

Supplementary Figure S8. Number of responses ranked within the top-5 per policy instrument category, regarding the influence on forest protection in the next 10 years, for the four spatial level subsamples. Note: the range of the x-axis varies between samples. Blank answers are not shown or considered for the total N in the graph.

5. Summary statistics for al variables across countries and across spatial scales

Supplementary Table S4. Summary statistics (count, average and standard deviation) of the studied variables for the total sample and the country subsamples.

	Total sample			Country								
				Zambia			Ecuador			Philippines		
**Variable**	**N**	**Average**	**Std. Dev.**	**N**	**Average**	**Std. Dev.**	**N**	**Average**	**Std. Dev.**	**N**	**Average**	**Std. Dev.**
Overall												
Alertness	218	19.92	11.54	71	13.88	7.49	65	24.23	12.14	82	21.73	11.91
(Commercial)		25.60	19.09		12.18	10.91		35.10	17.60		29.70	19.52
(Subsistence)		14.23	11.71		15.58	7.04		13.37	13.53		13.76	13.32
Confidence	217	28.74	16.23	71	19.14	14.30	64	33.22	13.86	82	33.57	16.00
Importance of (Drivers)	219			71			65			83		
Agriculture		43.36	24.11		40.07	21.27		53.80	21.83		37.99	25.72
Logging		15.24	17.05		10.05	15.19		18.02	18.00		17.49	16.99
Woodfuel		12.68	20.17		34.13	21.00		0.00	0.00		4.25	10.35
OilMining		10.67	16.15		5.04	10.23		14.94	15.55		12.14	19.26
Infrastructure		10.15	16.36		6.70	12.59		7.78	11.78		14.94	20.73
Plantations		2.15	6.70		1.13	5.49		3.38	8.34		2.05	6.10
Natural		4.99	12.28		0.75	2.53		1.77	6.28		11.13	17.40
OtherDrivers		0.79	4.99		2.14	8.31		0.31	2.48		0.00	0.00
Importance of (Policy instruments)	203			71			62			70		
Reforestation		38.02	26.27		45.42	29.33		26.13	19.41		41.04	24.94
ProtectedAreas		19.20	22.25		4.65	13.40		28.63	21.79		25.60	22.54
AntiLogging		16.30	22.11		16.62	23.22		14.19	21.16		17.83	21.94
Financial		9.84	17.33		3.24	12.76		24.92	19.91		3.19	8.37
LandUseRights		9.07	15.92		11.27	16.71		5.00	9.50		10.44	18.90
OtherPolicies		7.58	17.13		18.80	23.97		1.13	5.76		1.90	6.85

Supplementary Table S5. Summary statistics (count, average and standard deviation) of the studied variables for the spatial level subsamples.

	**Spatial level**											
	**International**			**National**			**Regional**			**Local**		
**Variable**	**N**	**Average**	**Std. Dev.**	**N**	**Average**	**Std. Dev.**	**N**	**Average**	**Std. Dev.**	**N**	**Average**	**Std. Dev.**
Overall												
Alertness	18	26.78	14.68	78	23.95	11.38	71	17.48	10.09	51	14.72	9.20
(Commercial)		36.49	21.65		31.53	17.63		21.69	19.38		18.15	15.54
(Subsistence)		17.07	15.20		16.38	12.38		13.27	10.63		11.29	10.08
Confidence	18	34.42	18.04	77	33.36	15.49	71	24.33	16.45	51	25.92	14.25
Importance of (Drivers)	18			79			71			51		
Agriculture		45.94	28.59		43.01	24.03		42.46	21.40		44.22	26.63
Logging		14.78	14.09		15.49	17.32		12.56	16.39		18.73	18.27
Woodfuel		14.17	19.87		6.56	14.58		16.87	20.02		15.78	25.52
OilMining		8.67	11.26		13.10	17.27		12.75	17.79		4.71	11.51
Infrastructure		14.50	19.43		12.67	17.18		8.24	14.88		7.35	15.41
Plantations		1.94	5.72		2.66	7.15		1.41	6.39		2.45	6.81
Natural		0.00	0.00		5.91	11.97		4.23	13.59		6.37	12.73
OtherDrivers		0.00	0.00		0.59	3.58		1.48	7.53		0.39	2.80
Importance of (Policy instruments)	17			72			67			47		
Reforestation		28.24	22.70		36.24	26.77		39.16	27.19		42.66	24.91
ProtectedAreas		22.35	18.88		21.88	22.85		14.96	21.43		20.00	23.31
AntiLogging		18.24	22.15		16.94	23.05		16.61	23.11		14.15	19.54
Financial		14.41	18.36		13.51	20.21		6.72	15.56		7.02	13.17
LandUseRights		9.12	15.43		9.17	17.64		8.52	14.22		9.68	16.06
OtherPolicies		7.65	17.86		2.26	7.71		14.03	21.89		6.49	17.10

6. Variables and transformations: analyzing univariate and multivariate normality

Supplementary Table S6. Skewness, histograms, boxplots, Shapiro-Wilk and Mardia normality results for the variables related to drivers of deforestation, before and after transformation (we selected the method which brought skewness the closest to zero, between square-root, log or inverse).

Variable		Transf.	Skewness	Histogram	Boxplot	Shapiro-Wilk (univ.)	Mardia (multiv.)
***Alertness* (total)**	Before		0.8151833	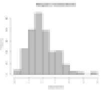	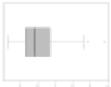	W = 0.95496,<br>p-value <0.0001	Mardia tests with all the driver-related variables:<br><br>Skewness Statistic: -516,871.4657 p-value 1 Result: YES<br><br>Kurtosis Statistic: 2,336.668 p-value 0 Result: NO<br><br>Multivariate normality Result: NO
	After	Square root positive sqrt(x+1)	-0.0533145	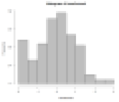	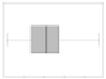	W = 0.97239,<br>p-value <0.001	
***Alertness* (commercial)**	Before		0.5092041	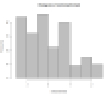	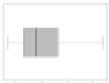	W = 0.94115,<br>p-value <0.0001	
	After	Square root positive sqrt(x+1)	-0.09711656	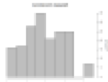	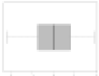	W = 0.97179,<br>p-value <0.01	
***Alertness* (subsistence)**	Before		1.340764	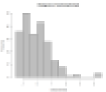	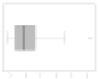	W = 0.89248,<br>p-value <0.0001	
	After	Log positive (log(1+x1))	-0.08466224	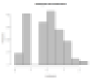	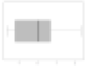	W = 0.94401,<br>p-value <0.0001	
Agriculture	Before		-0.08972876			W = 0.96548,<br>p-value <0.0001	
	After	None		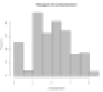	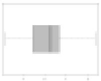		
Logging	Before		1.204512	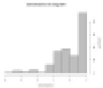	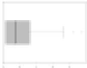	W = 0.83054,<br>p-value <0.0001	
	After	Log positive (log(1+x1))	-0.1107971	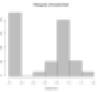	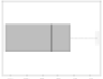	W = 0.80097,<br>p-value <0.0001	

…continues…

… Supplementary Table S6 continues …

Woodfuel	Before		1.525409	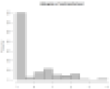	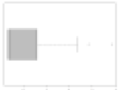	W = 0.68331, p-value <0.0001	Mardia tests with all the driver-related and policy-related variables:<br><br>Skewness Statistic: -830.4816 p-value 0 Result: NO<br><br>Kurtosis Statistic: 10.2107 p-value 0 Result: NO<br><br>Multivariate normality Result: NO
	After	Inverse positive 1/(x+1)	-0.6680042	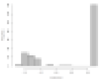	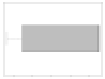	W = 0. 65115, p-value <0.0001	
OilMining	Before		1.672852	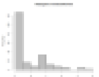	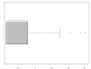	W = 0.71021, p-value <0.0001	
	After	Inverse positive 1/(x+1)	-0.4234723	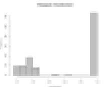	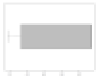	W = 0.69099, p-value <0.0001	
Infrastructure	Before		1.765333	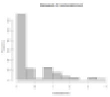	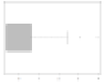	W = 0.682, p-value <0.0001	
	After	Inverse positive 1/(x+1)	-0.5743924	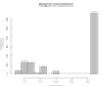	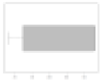	W = 0.68622, p-value <0.0001	
Plantations	Before		3.273199	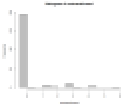		W = 0.36196, p-value <0.0001	
	After	Inverse positive 1/(x+1)	-2.554486	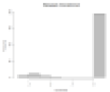		W = 0.36985, p-value <0.0001	
Natural	Before		2.813872	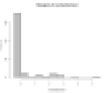		W = 0.47266, p-value <0.0001	
	After	Inverse positive 1/(x+1)	-1.692043	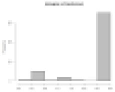		W = 0.50676, p-value <0.0001	
OtherDrivers	Before		7.220588	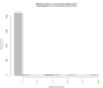		W = 0.14776, p-value <0.0001	
	After	Inverse positive 1/(x+1)	-5.645995	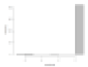		W = 0.16399, p-value <0.0001	

Supplementary Table S7. Skewness, histograms, boxplots, Shapiro-Wilk and Mardia normality results for the variables related to policy instruments, before and after transformation (we selected the method which brought skewness the closest to zero, between square-root, log or inverse).

Variable		Transf.	Skewness	Histogram	Boxplot	Shapiro-Wilk (univ.)	Mardia (multiv.)
Confidence	Before		0.2008255	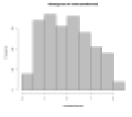	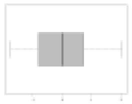	W = 0.96836, p-value <0.0001	Mardia tests with all the policy-related variables:<br><br>Skewness Statistic: -516,871.4657 p-value 1 Result: YES<br><br>Kurtosis Statistic: 2,336.668 p-value 0 Result: NO<br><br>Multivariate normality Result: NO
	After	None					
Reforestation	Before		0.3028384	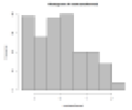	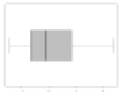	W = 0.95392, p-value <0.0001	
	After	Square root positive sqrt(x+1)	-0.0013587	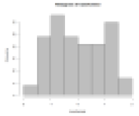	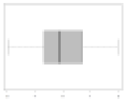	W = 0.95781, p-value <0.0001	
ProtectedAreas	Before		0.933102	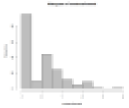	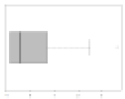	W = 0.81944, p-value <0.0001	
	After	Log positive (log(1+x1))	0.08156469	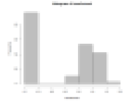	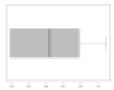	W = 0.77097, p-value <0.0001	
AntiLogging	Before		1.492621	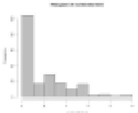	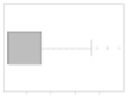	W = 0.76399, p-value <0.0001	
	After	Inverse positive 1/(x+1)	-0.1035551	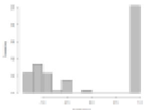	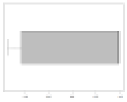	W = 0.72234, p-value <0.0001	

…continues…

… Supplementary Table S7 continues …

Financial	Before		1.844748	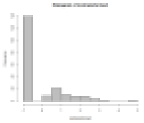	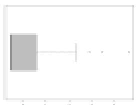	W = 0.63772, p-value <0.0001
	After	Inverse positive 1/(x+1)	-0.8742986	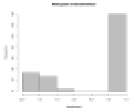	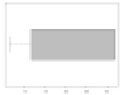	W = 0.62367, p-value <0.0001
LandUseRights	Before		2.166816	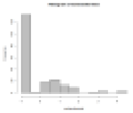	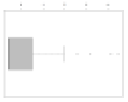	W = 0.6374, p-value <0.0001
	After	Inverse positive 1/(x+1)	-0.738239	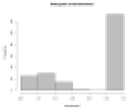	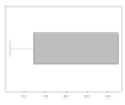	W = 0.65711, p-value <0.0001
OtherPolicies	Before		2.469109	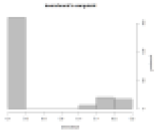	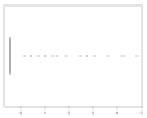	W = 0.51509, p-value <0.0001
	After	Inverse positive 1/(x+1)	-1.492949	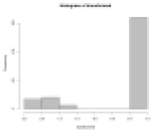	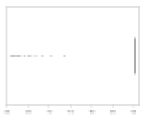	W = 0.53099, p-value <0.0001

7. Principal component analysis (PCA)

(a)

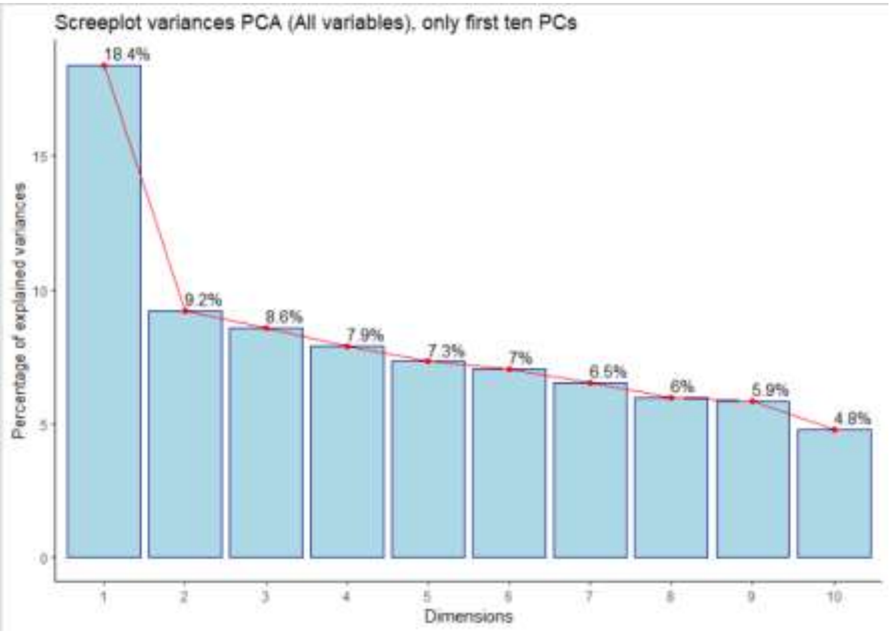

(b)

	sdev	eigenv	var	cumvar
**PC1**	1.82	3.31	18.39	18.39
**PC2**	1.29	1.66	9.22	27.61
**PC3**	1.24	1.55	8.58	36.20
**PC4**	1.19	1.42	7.90	44.10
**PC5**	1.15	1.32	7.32	51.42
**PC6**	1.13	1.27	7.03	58.45
**PC7**	1.08	1.17	6.53	64.98
**PC8**	1.04	1.07	5.97	70.95
**PC9**	1.03	1.06	5.86	76.81
**PC10**	0.93	0.86	4.77	81.58
**PC11**	0.92	0.84	4.68	86.26
**PC12**	0.89	0.79	4.36	90.63
**PC13**	0.81	0.66	3.65	94.27
**PC14**	0.76	0.58	3.22	97.49
**PC15**	0.67	0.45	2.51	100.00
**PC16**	0.00	0.00	0.00	100.00
**PC17**	0.00	0.00	0.00	100.00
**PC18**	0.00	0.00	0.00	100.00

(c)

		PC1	PC2	PC3	PC4	PC5	PC6	PC7	PC8	PC9	PC10	PC11	PC12	PC13	PC14	PC15	PC16	PC17	PC18
**Country**	**Zambia**	1.80	0.52	0.15	0.12	-0.12	-0.14	0.18	-0.05	-0.06	0.14	-0.04	-0.05	0.15	0.07	0.05	0.00	0.00	0.00
	**Ecuador**	-1.33	-0.24	0.55	-0.16	-0.05	0.62	0.04	-0.07	0.19	-0.01	0.03	-0.10	0.11	-0.08	-0.06	0.00	0.00	0.00
	**Philippines**	-0.65	-0.32	-0.65	0.02	0.17	-0.41	-0.22	0.11	-0.11	-0.13	0.01	0.15	-0.26	0.00	0.01	0.00	0.00	0.00
**Spatial level**	**International**	-0.80	0.66	0.41	-0.12	0.06	-0.09	0.07	0.10	0.05	0.12	-0.14	-0.07	-0.11	0.26	0.03	0.00	0.00	0.00
	**National**	-0.79	0.02	-0.15	-0.07	0.02	-0.09	-0.06	-0.06	0.15	0.03	0.06	-0.04	0.06	0.02	-0.01	0.00	0.00	0.00
	**Regional**	0.64	0.03	0.10	-0.07	-0.14	-0.02	0.20	-0.14	-0.01	-0.05	-0.09	-0.01	-0.09	-0.14	-0.08	0.00	0.00	0.00
	**Local**	0.57	-0.32	-0.07	0.24	0.16	0.19	-0.21	0.25	-0.23	-0.03	0.08	0.10	0.07	0.09	0.11	0.00	0.00	0.00

Supplementary Figure S9. Results of the PCA with all the variables: spree plot (a) showing the percentage of explained variances of the ten first principal components, table (b) listing the standard deviation (sdev), eigenvalue (eigenv), variance (var) and cumulative variance (cumvar) of each principal component and table (c) listing the average score of each principal component, grouped by country and spatial level

(a)

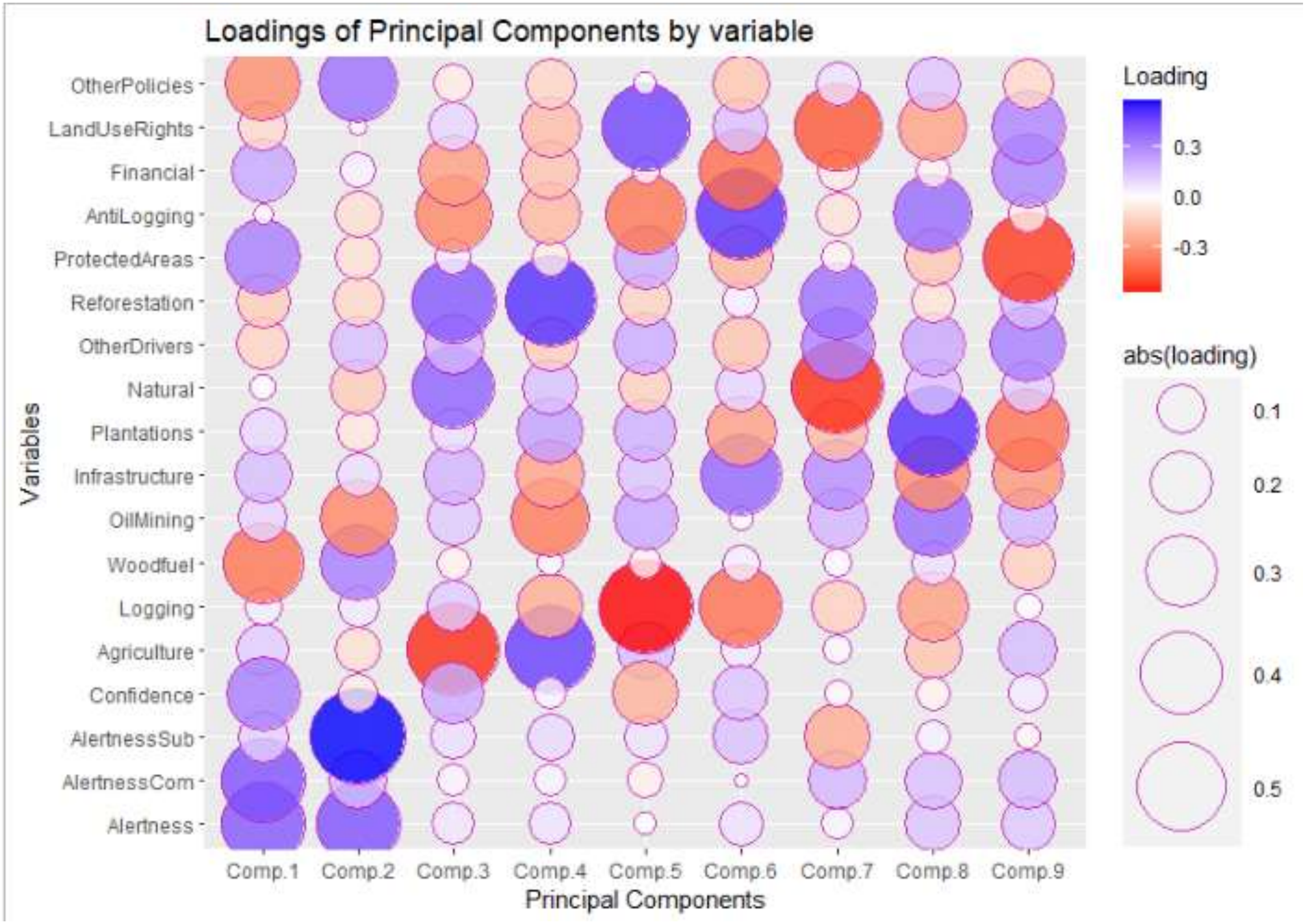

Supplementary Figure S10. Results of the PCA: correlation plot (a) and table (b) showing the loadings of the different principal components for each variable. Signs have to be reversed for interpretation. (…continues…)

	PC1	PC2	PC3	PC4	PC5	PC6	PC7	PC8	PC9	PC10	PC11	PC12	PC13	PC14	PC15	PC16	PC17	PC18
Overall																		
Alertness	0.42	0.43	0.07	0.07	0.00	0.08	0.03	0.15	0.14	0.14	0.09	0.07	0.15	0.04	0.08	0.02	0.00	0.72
AlertnessCom	0.44	0.17	0.03	0.03	-0.04	0.00	0.18	0.16	0.17	0.21	-0.03	0.09	0.48	-0.16	0.18	-0.02	0.00	-0.59
AlertnessSub	0.12	0.58	0.08	0.09	0.07	0.15	-0.24	0.03	-0.01	-0.07	0.23	0.00	-0.48	0.35	-0.12	-0.01	0.00	-0.36
Confidence	0.32	-0.05	0.21	0.03	-0.22	0.14	-0.01	-0.03	0.05	-0.32	0.13	-0.33	-0.18	-0.63	-0.35	0.00	0.00	0.00
Importance (Drivers)																		
Agriculture	0.12	-0.09	-0.54	0.48	0.16	0.05	0.02	-0.17	0.17	-0.20	0.06	0.10	0.07	0.03	-0.18	0.48	0.23	-0.02
Logging	0.04	0.06	0.13	-0.23	-0.57	-0.40	-0.13	-0.28	0.01	0.13	0.12	0.39	-0.03	0.02	-0.13	0.35	0.16	-0.01
Woodfuel	-0.39	0.32	-0.03	0.01	-0.03	0.04	0.02	0.08	-0.13	0.15	0.13	-0.29	-0.06	-0.38	0.48	0.42	0.20	-0.01
OilMining	0.10	-0.34	0.13	-0.38	0.22	0.01	0.18	0.37	0.18	0.11	0.47	-0.17	-0.06	0.23	-0.11	0.33	0.16	-0.01
Infrastructure	0.16	0.08	0.19	-0.26	0.14	0.38	0.28	-0.33	-0.30	0.11	-0.48	-0.08	-0.01	0.13	-0.16	0.32	0.15	-0.01
Plantations	0.09	-0.07	0.08	0.24	0.20	-0.28	-0.20	0.51	-0.40	0.29	-0.27	0.17	-0.16	-0.20	-0.27	0.13	0.06	0.00
Natural	0.01	-0.15	0.40	0.15	-0.13	0.10	-0.54	0.16	0.13	-0.30	-0.25	-0.19	0.27	0.26	0.18	0.23	0.11	-0.01
OtherDrivers	-0.12	0.16	0.18	-0.14	0.21	-0.16	0.32	0.22	0.32	-0.52	-0.27	0.40	-0.19	-0.14	0.12	0.10	0.05	0.00
Importance (Policies)																		
Reforestation	-0.14	-0.11	0.42	0.51	-0.13	0.04	0.35	-0.08	0.16	0.23	0.05	0.01	-0.13	0.09	-0.01	0.22	-0.47	-0.01
ProtectedAreas	0.32	-0.09	0.04	-0.04	0.21	-0.21	-0.03	-0.16	-0.52	-0.35	0.25	0.08	0.04	0.01	0.34	0.19	-0.40	-0.01
AntiLogging	0.00	-0.09	-0.34	-0.20	-0.39	0.51	-0.07	0.37	-0.05	-0.03	-0.09	0.25	-0.11	-0.02	0.06	0.19	-0.40	-0.01
Financial	0.22	0.04	-0.28	-0.17	-0.01	-0.41	-0.06	-0.03	0.31	0.11	-0.38	-0.46	-0.27	0.08	0.09	0.15	-0.31	0.00
LandUseRights	-0.10	0.00	0.10	-0.19	0.47	0.15	-0.46	-0.27	0.31	0.24	0.06	0.24	0.07	-0.31	-0.09	0.14	-0.29	0.00
OtherPolicies	-0.33	0.36	-0.05	-0.12	0.01	-0.16	0.07	0.14	-0.11	-0.19	0.05	-0.19	0.48	0.07	-0.50	0.15	-0.31	0.00

Supplementary Figure S10 (continuation). Results of the PCA: correlation plot (a) and table (b) showing the loadings of the different principal components for each variable. Signs have to be reversed for interpretation.

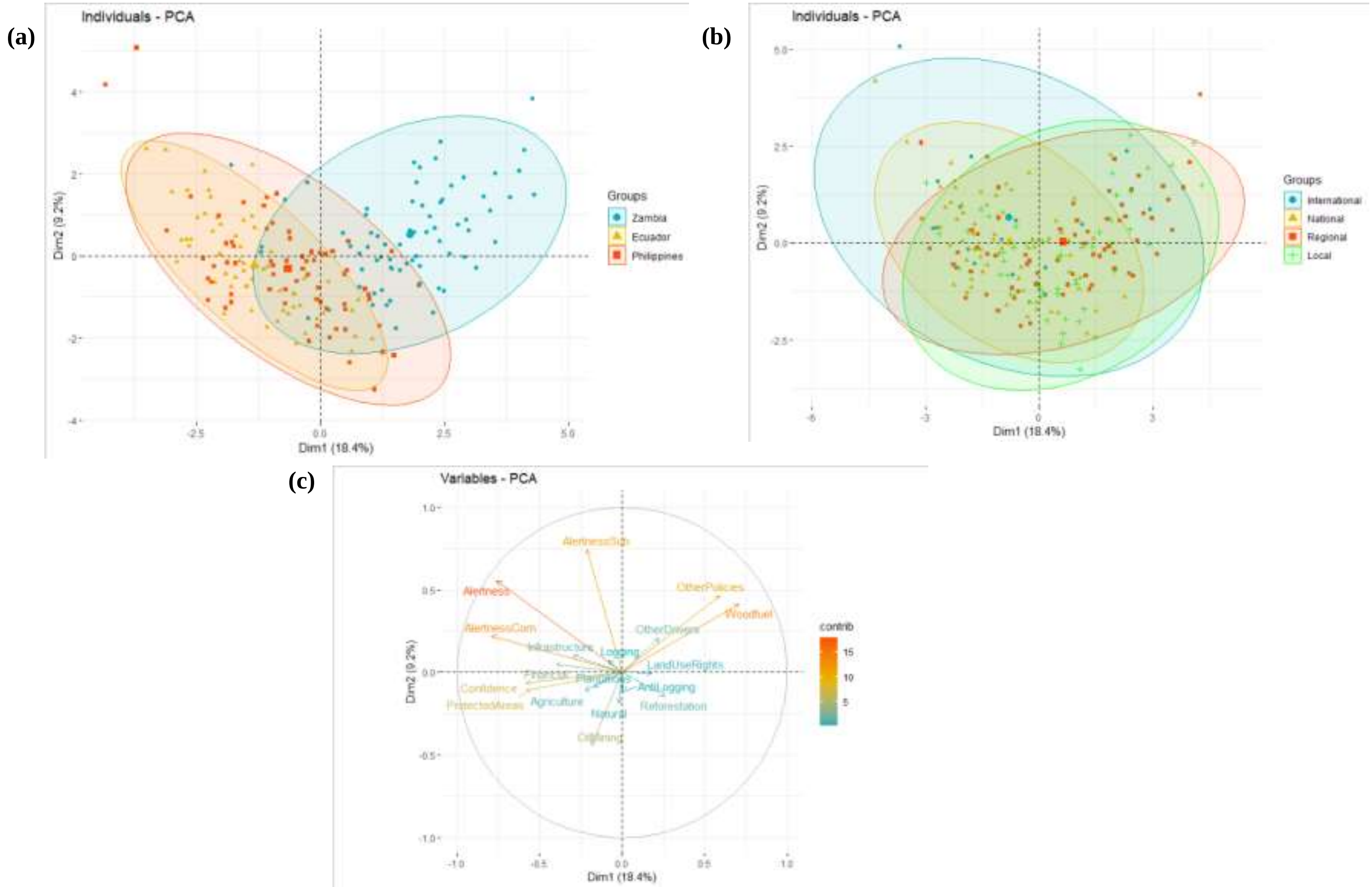

Supplementary Figure S11. Results of the PCA with all the variables: biplots of the individuals grouped by country (a) and spatial level (b) and of the variables (driver categories) (c) for the two first components.

8. Non-parametric analysis of variance: one-way Kruskal-Wallis and Dunn tests

Supplementary Table S8. Results of the Kruskal-Wallis and Dunn tests for all driver-related variables across countries and spatial levels, including significance (sign, ****: <0.0001, ***: <0.001, **: <0.01, *: <0.05, ns: not significant [>0.05]) total and cross-groups (Zmb: Zambia, Ecu: Ecuador, Phl: Philippines Int: International, Nat: National, Reg: Regional, Loc: Local), degrees of freedom (df), chi square statistic (χ2), effect size partial eta squared (effsize) and magnitude (magn, lar: large, mod: moderate, sma: small). See Figure S22.

			Alertness			*Agriculture*	*Logging*	*Woodfuel*	*OilMining*	*Infrastructure*	*Plantations*	*Natural*	*OtherDrivers*
			Tot	*Com*	*Sub*								
		**N**	218	218	218	219	219	219	219	219	219	219	219
**Countries (df=2)**	**Kruskal-Wallis**	**sign**	****	****	ns	***	**	****	***	ns	ns	****	**
		**χ2**	37.9	64.3	4.99	14.5	12.0	140.0	17.4	4.35	5.46	25.7	9.65
		**effsize**	0.17	0.29	0.01	0.06	0.05	0.639	0.07	0.01	0.02	0.11	0.04
		**magn**	lar	lar	sma	sma	sma	lar	mod	sma	sma	mod	sma
	**Dunn test**	**Zmb - Ecu**	****	****	ns	**	**	****	****	ns	ns	ns	ns
		**Zmb - Phl**	****	****	ns	ns	**	****	ns	ns	ns	****	**
		**Ecu - Phl**	ns	ns	ns	**	ns	ns	ns	ns	ns	***	ns
**Spatial levels (df=3)**	**Kruskal-Wallis**	**sign**	****	****	ns	ns	ns	**	**	ns	ns	ns	ns
		**χ2**	29.2	25.2	6.83	0.38	3.92	12.7	13.3	7.13	2.98	7.74	1.20
		**effsize**	0.12	0.10	0.02	-0.01	0.01	0.05	0.05	0.02	-0.01	0.02	-0.01
		**magn**	mod	mod	sma	sma	sma	sma	sma	sma	sma	sma	sma
	**Dunn test**	**Int - Nat**	ns	ns	ns	ns	ns	ns	ns	ns	ns	ns	ns
		**Int - Reg**	*	*	ns	ns	ns	ns	ns	ns	ns	ns	ns
		**Int - Loc**	**	**	ns	ns	ns	ns	ns	ns	ns	ns	ns
		**Nat - Reg**	**	**	ns	ns	ns	**	ns	ns	ns	ns	ns
		**Nat - Loc**	****	***	ns	ns	ns	ns	**	ns	ns	ns	ns
		**Reg - Loc**	ns	ns	ns	ns	ns	ns	**	ns	ns	ns	ns

Supplementary Table S9. Results of the Kruskal-Wallis and Dunn tests for all the policy-related variables across countries and spatial levels, including significance (sign, ****: <0.0001, ***: <0.001, **: <0.01, *: <0.05, ns: not significant [>0.05]) total and cross-groups (Zmb: Zambia, Ecu: Ecuador, Phl: Philippines, Int: International, Nat: National, Reg: Regional, Loc: Local), chi square statistic (χ2), effect size partial eta squared (effsize) and magnitude (magn, lar: large, mod: moderate, sma: small). See Figure S22.

			Confidence	***Reforestation***	***ProtectedAreas***	***AntiLogging***	***Financial***	***LandUseRights***	***OtherPolicies***
		**N**	217	203	203	203	203	203	203
**Countries (df=2)**	**Kruskal-Wallis**	**sign**	****	****	****	ns	****	ns	****
		**χ2**	40.0	19.5	58.6	1.17	84.0	5.11	49.4
		**effsize**	0.18	0.09	0.28	-0.01	0.41	0.02	0.24
		**magn**	lar	mod	lar	sma	lar	sma	lar
	**Dunn test**	**Zmb - Ecu**	****	****	****	ns	****	ns	****
		**Zmb - Phl**	****	ns	****	ns	ns	ns	****
		**Ecu - Phl**	ns	**	ns	ns	****	ns	ns
**Spatial levels (df=3)**	**Kruskal-Wallis**	**sign**	**	ns	ns	ns	ns	ns	***
		**χ2**	16.0	3.83	5.55	0.33	7.30	0.42	19.2
		**effsize**	0.06	0.01	0.01	0.01	0.02	-0.01	0.08
		**magn**	mod	sma	sma	sma	sma	sma	mod
	**Dunn test**	**Int - Nat**	ns	ns	ns	ns	ns	ns	ns
		**Int - Reg**	ns	ns	ns	ns	ns	ns	ns
		**Int - Loc**	ns	ns	ns	ns	ns	ns	ns
		**Nat - Reg**	**	ns	ns	ns	ns	ns	***
		**Nat - Loc**	ns	ns	ns	ns	ns	ns	ns
		**Reg - Loc**	ns	ns	ns	ns	ns	ns	*

9. Parametric analysis of variance: one-way ANOVA and Tukey multiple comparison of means

Supplementary Table S10. Results of the parametric one-way ANOVA and Tukey tests for all driver-related variables across countries and spatial levels, including significance (sign, ****: <0.0001, ***: <0.001, **: <0.01, *: <0.05, ns: not significant [>0.05]) overall and cross-groups (Zmb: Zambia, Ecu: Ecuador, Phl: Philippines, Int: International, Nat: National, Reg: Regional, Loc: Local), degrees of freedom (df), sum and mean of squares (SumSq, MeanSq), F-Values, mean differences (diff.) and confidence intervals (lower, upper). (…continues…)

Variable	Parametric one-way ANOVA (transformed variables)						Tukey's HSD (honestly significant difference) test									
		**df**	**SumSq**	**MeanSq**	**F-value**	**Pr(>F)**	**pair**	**diff.**	**lower**	**upper**	**p-adj**	**pair**	**diff.**	**lower**	**upper**	**p-adj**
Alertness	**Country**	2	47.58	23.79	31.4	****	**Ecu-Zmb**	1.1317	0.7563	1.5072	****					
	**Residuals**	188	142.42	0.76			**Phl-Zmb**	1.0097	0.6507	1.3686	****	**Loc-Int**	-0.5432	-1.2563	0.1699	ns
							**Phl-Ecu**	-0.1221	-0.4843	0.2402	ns	**Reg-Nat**	-0.6576	-1.0844	-0.2309	***
	**Spatial level**	3	18.65	6.217	6.784	***	**Nat-Int**	0.04649	-0.6065	0.6995	ns	**Loc-Nat**	-0.5897	-1.0905	-0.0890	*
	**Residuals**	187	171.35	0.916			**Reg-Int**	-0.6112	-1.2743	0.0520	ns	**Loc-Reg**	0.0679	-0.4460	0.5819	ns
Alertness (comm.)	**Country**	2	31.23	15.616	18.84	****	**Ecu-Zmb**	1.1438	0.7029	1.5847	****					
	**Residuals**	171	141.77	0.829			**Phl-Zmb**	0.7141	0.2927	1.1356	***	**Loc-Int**	-1.0524	-1.7849	-0.3198	**
							**Phl-Ecu**	-0.4297	-0.8043	-0.0550	*	**Reg-Nat**	-0.2772	-0.7383	0.1839	ns
	**Spatial level**	3	19.25	6-416	7.049	***	**Nat-Int**	-0.2892	-0.9721	0.3937	ns	**Loc-Nat**	-0.7632	-1.2550	-0.2714	***
	**Residuals**	170	153.75	0.904			**Reg-Int**	-0.5664	-1.2787	0.1459	ns	**Loc-Reg**	-0.4860	-1.0179	0.0460	ns
Alertness (subs.)	**Country**	2	0.4	0.20	0.198	ns	**Ecu-Zmb**	-0.0613	-0.5075	0.3849	ns					
	**Residuals**	179	180.6	1.01			**Phl-Zmb**	-0.1089	-0.5193	0.3014	ns	**Loc-Int**	-0.1551	-0.9383	0.6281	ns
							**Phl-Ecu**	-0.0476	-0.4994	0.4041	ns	**Reg-Nat**	-0.1692	-0.6277	0.2894	ns
	**Spatial level**	3	1.19	0.398	0.394	ns	**Nat-Int**	-0.0394	-0.7617	0.6829	ns	**Loc-Nat**	-0.1157	-0.6503	0.4189	ns
	**Residuals**	178	179.81	1.01			**Reg-Int**	-0.2085	-0.9419	0.5249	ns	**Loc-Reg**	0.0534	-0.4961	0.6029	ns
Agricul-ture	**Country**	2	17.63	8.817	9.506	***	**Ecu-Zmb**	0.5695	0.1793	0.9597	**					
	**Residuals**	216	200.37	0.928			**Phl-Zmb**	-0.0864	-0.4538	0.2811	ns	**Loc-Int**	-0.0717	-0.7859	0.6425	ns
							**Phl-Ecu**	-0.6559	-1.0324	-0.2795	***	**Reg-Nat**	-0.0227	-0.4487	0.4033	ns
	**Spatial level**	3	0.39	0.1285	0.127	ns	**Nat-Int**	-0.1216	-0.8020	0.5587	ns	**Loc-Nat**	0.0499	-0.4180	0.5178	ns
	**Residuals**	215	217.65	1.0122			**Reg-Int**	-0.1443	-0.8318	0.5431	ns	**Loc-Reg**	0.0726	-0.4055	0.5508	ns

Supplementary Table S10 (continuation)**.** Results of the parametric one-way ANOVA and Tukey tests for all driver-related variables across countries and spatial levels, including significance (sign, ****: <0.0001, ***: <0.001, **: <0.01, *: <0.05, ns: not significant [>0.05]) overall and cross-groups (Zmb: Zambia, Ecu: Ecuador, Phl: Philippines, Int: International, Nat: National, Reg: Regional, Loc: Local), degrees of freedom (df), sum and mean of squares (SumSq, MeanSq), F-Values, mean differences (diff.) and confidence intervals (lower, upper). (…continues…)

Variable	Parametric one-way ANOVA (transformed variables)						Tukey's HSD (honestly significant difference) test									
		**df**	**SumSq**	**MeanSq**	**F-value**	**Pr(>F)**	**pair**	**diff.**	**lower**	**upper**	**p-adj**	**pair**	**diff.**	**lower**	**upper**	**p-adj**
Logging	**Country**	2	10.44	5.22	5.432	**	**Ecu-Zmb**	0.4905	0.0934	0.8877	*					
	**Residuals**	216	207.56	0.961			**Phl-Zmb**	0.4445	0.0706	0.8185	*	**Loc-Int**	0.1674	-0.5411	0.8760	ns
							**Phl-Ecu**	-0.0460	-0.4292	0.3372	ns	**Reg-Nat**	-0.1254	-0.5480	0.2972	ns
	**Spatial level**	3	3.81	1.2692	1.274	ns	**Nat-Int**	-0.0623	-0.7373	0.6127	ns	**Loc-Nat**	0.2297	-0.2345	0.6940	ns
	**Residuals**	215	214.19	0.9962			**Reg-Int**	-0.1877	-0.8697	0.4943	ns	**Loc-Reg**	0.3552	-0.1192	0.8295	ns
Woodfuel	**Country**	2	136.86	68.43	182.2	****	**Ecu-Zmb**	1.8551	1.6068	2.1034	****					
	**Residuals**	216	81.14	0.38			**Phl-Zmb**	1.5108	1.2769	1.7446	****	**Loc-Int**	0.0715	-0.6217	0.7647	ns
							**Phl-Ecu**	-0.3443	-0.5839	-0.1048	**	**Reg-Nat**	-0.5839	-0.9973	-0.1704	**
	**Spatial level**	3	12.98	4.327	4.538	**	**Nat-Int**	0.3887	-0.2717	1.0491	ns	**Loc-Nat**	-0.3172	-0.7714	0.1370	ns
	**Residuals**	215	205.02	0.954			**Reg-Int**	-0.1952	-0.8624	0.4721	ns	**Loc-Reg**	0.2667	-0.1974	0.7308	ns
Oil	**Country**	2	17.53	8.767	9.447	***	**Ecu-Zmb**	-0.7135	-1.1037	-0.3232	***					
Mining	**Residuals**	216	200.47	0.926			**Phl-Zmb**	-0.2698	-0.6373	0.0977	ns	**Loc-Int**	0.4735	-0.2195	1.1666	ns
							**Phl-Ecu**	0.4437	0.0671	0.8202	*	**Reg-Nat**	0.0180	-0.3954	0.4314	ns
	**Spatial level**	3	13.06	4.353	4.567	**	**Nat-Int**	-0.1199	-0.7802	0.5403	ns	**Loc-Nat**	0.5935	0.1394	1.0476	**
	**Residuals**	215	204.94	0.953			**Reg-Int**	-0.1020	-0.7691	0.5651	ns	**Loc-Reg**	0.5755	0.1115	1.0395	**
Infrastr-	**Country**	2	2.89	1.4445	1.45	ns	**Ecu-Zmb**	-0.0923	-0.4966	0.3120	ns					
ucture	**Residuals**	216	215.11	0.9959			**Phl-Zmb**	-0.2687	-0.6494	0.1120	ns	**Loc-Int**	0.4812	-0.2216	1.1840	ns
							**Phl-Ecu**	-0.1764	-0.5664	0.2137	ns	**Reg-Nat**	0.2744	-0.1448	0.6936	ns
	**Spatial level**	3	7.28	2.4256	2.475	ns	**Nat-Int**	0.0549	-0.6146	0.7243	ns	**Loc-Nat**	0.4263	-0.0341	0.8868	ns
	**Residuals**	215	210.72	0.9801			**Reg-Int**	0.3293	-0.3472	1.0057	ns	**Loc-Reg**	0.1519	-0.3186	0.6224	ns

Supplementary Table S10 (continuation)**.** Results of the parametric one-way ANOVA and Tukey tests for all driver-related variables across countries and spatial levels, including significance (sign, ****: <0.0001, ***: <0.001, **: <0.01, *: <0.05, ns: not significant [>0.05]) overall and cross-groups (Zmb: Zambia, Ecu: Ecuador, Phl: Philippines, Int: International, Nat: National, Reg: Regional, Loc: Local), degrees of freedom (df), sum and mean of squares (SumSq, MeanSq), F-Values, mean differences (diff.) and confidence intervals (lower, upper).

Variable	Parametric one-way ANOVA (transformed variables)						Tukey's HSD (honestly significant difference) test									
		**df**	**SumSq**	**MeanSq**	**F-value**	**Pr(>F)**	**pair**	**diff.**	**lower**	**upper**	**p-adj**	**pair**	**diff.**	**lower**	**upper**	**p-adj**
Plantation	**Country**	2	5.4	2.6977	2.741	ns	**Ecu-Zmb**	-0.3964	-0.7984	0.0055	ns					
	**Residuals**	216	212.6	0.9843			**Phl-Zmb**	-0.2242	-0.6027	0.1543	ns	**Loc-Int**	-0.0678	-0.7778	0.6423	ns
							**Phl-Ecu**	0.1722	-0.2156	0.5600	ns	**Reg-Nat**	0.2574	-0.1662	0.6809	ns
	**Spatial level**	3	2.87	0.9559	0.955	ns	**Nat-Int**	-0.0883	-0.7648	0.5881	ns	**Loc-Nat**	0.0206	-0.4447	0.4858	ns
	**Residuals**	215	215.13	1.0006			**Reg-Int**	0.1690	-0.5145	0.8525	ns	**Loc-Reg**	-0.2368	-0.7122	0.2386	ns
Natural	**Country**	2	29.07	14.535	16.62	****	**Ecu-Zmb**	-0.0779	-0.4568	0.3010	ns					
	**Residuals**	216	188.93	0.875			**Phl-Zmb**	-0.7856	-1.1424	-0.4288	****	**Loc-Int**	-0.6335	-1.3357	0.0686	ns
							**Phl-Ecu**	-0.7076	-1.0732	-0.3421	****	**Reg-Nat**	0.2480	-0.1709	0.6668	ns
	**Spatial level**	3	7.65	2.5488	2.605	ns	**Nat-Int**	-0.5980	-1.2669	0.0709	ns	**Loc-Nat**	-0.0355	-0.4956	0.4245	ns
	**Residuals**	215	210.35	0.9784			**Reg-Int**	-0.3501	-1.0259	0.3258	ns	**Loc-Reg**	-0.2835	-0.7536	0.1866	ns
Other	**Country**	2	8.82	4.409	4.552	*	**Ecu-Zmb**	0.3713	-0.0274	0.7700	ns					
Drivers	**Residuals**	216	209.18	0.968			**Phl-Zmb**	0.4608	0.0854	0.8363	*	**Loc-Int**	-0.1141	-0.8268	0.5986	ns
							**Phl-Ecu**	0.0895	-0.2951	0.4742	ns	**Reg-Nat**	-0.0833	-0.5084	0.3418	ns
	**Spatial level**	3	1.28	0.4262	0.423	ns	**Nat-Int**	-0.1787	-0.8577	0.5002	ns	**Loc-Nat**	0.0646	-0.4023	0.5316	ns
	**Residuals**	215	216.72	1.008			**Reg-Int**	-0.2620	-0.9480	0.4240	ns	**Loc-Reg**	0.1479	-0.3293	0.6251	ns

Supplementary Table S11. Results of the parametric one-way ANOVA and Tukey tests for all policy-related variables across countries and spatial levels, including significance (sign, ****: <0.0001, ***: <0.001, **: <0.01, *: <0.05, ns: not significant [>0.05]) overall and cross-groups (Zmb: Zambia, Ecu: Ecuador, Phl: Philippines, Int: International, Nat: National, Reg: Regional, Loc: Local), degrees of freedom (df), sum and mean of squares (SumSq, MeanSq), F-Values, mean differences (diff.) and confidence intervals (lower, upper). (…continues…)

Variable	Parametric one-way ANOVA (transformed variables)						Tukey's HSD (honestly significant difference) test									
		**df**	**SumSq**	**MeanSq**	**F-value**	**Pr(>F)**	**pair**	**diff.**	**lower**	**upper**	**p-adj**	**pair**	**diff.**	**lower**	**upper**	**p-adj**
Confid-	**Country**	2	36.96	18.481	22.09	****	**Ecu-Zmb**	0.8673	0.4952	1.2394	****					
ence	**Residuals**	214	179.04	0.837			**Phl-Zmb**	0.8889	0.5389	1.2388	****	**Loc-Int**	-0.5239	-1.2131	0.1653	ns
							**Phl-Ecu**	0.0216	-0.3385	0.3816	ns	**Reg-Nat**	-0.5568	-0.9705	-0.1432	**
	**Spatial level**	3	15.25	5.083	5.393	**	**Nat-Int**	-0.0650	-0.7232	0.5931	ns	**Loc-Nat**	-0.4589	-0.9127	-0.0050	*
	**Residuals**	213	200.75	0.942			**Reg-Int**	-0.6219	-1.2853	0.0415	ns	**Loc-Reg**	0.0980	-0.3634	0.5594	ns
Refores-	**Country**	2	25.71	12.854	15.07	****	**Ecu-Zmb**	-0.9828	-1.4141	-0.5515	****					
tation	**Residuals**	161	137.29	0.853			**Phl-Zmb**	-0.2783	-0.6806	0.1239	ns	**Loc-Int**	0.3066	-0.5480	1.1611	ns
							**Phl-Ecu**	0.7045	0.2764	1.1326	***	**Reg-Nat**	-0.0170	-0.5148	0.4809	ns
	**Spatial level**	3	1.2	0.4016	0.397	ns	**Nat-Int**	0.3409	-0.4909	1.1727	ns	**Loc-Nat**	-0.0343	-0.5693	0.5007	ns
	**Residuals**	160	161.8	1.0112			**Reg-Int**	0.3239	-0.5079	1.1557	ns	**Loc-Reg**	-0.0174	-0.5524	0.5176	ns
Protected	**Country**	2	65.92	32.96	48.44	****	**Ecu-Zmb**	1.2836	0.9450	1.6222	****					
Areas	**Residuals**	200	136.08	0.68			**Phl-Zmb**	1.0966	0.7685	1.4247	****	**Loc-Int**	-0.2400	-0.9673	0.4873	ns
							**Phl-Ecu**	-0.1870	-0.5267	0.1527	ns	**Reg-Nat**	-0.3633	-0.7995	0.0730	ns
	**Spatial level**	3	6.21	2.0711	2.105	ns	**Nat-Int**	-0.1456	-0.8386	0.5473	ns	**Loc-Nat**	-0.0943	-0.5763	0.3876	ns
	**Residuals**	199	195.79	0.9839			**Reg-Int**	-0.5089	-1.2068	0.1890	ns	**Loc-Reg**	0.2689	-0.2201	0.7579	ns
Anti	**Country**	2	1.3	0.6493	0.647	ns	**Ecu-Zmb**	0.0322	-0.3789	0.4434	ns		0.0456	-0.6926	0.7838	
Logging	**Residuals**	200	200.7	1.0035			**Phl-Zmb**	-0.1510	-0.5494	0.2474	ns	**Loc-Int**	0.08428	-0.3585	0.5270	ns
							**Phl-Ecu**	-0.1832	-0.5958	0.2293	ns	**Reg-Nat**	0.0878	-0.4013	0.5769	ns
	**Spatial level**	3	0.33	0.1088	0.107	ns	**Nat-Int**	-0.0422	-0.7455	0.6611	ns	**Loc-Nat**	0.0035	-0.4927	0.4998	ns
	**Residuals**	199	201.67	1.0134			**Reg-Int**	0.0421	-0.6662	0.7504	ns	**Loc-Reg**	0.0456	-0.6926	0.7838	ns

Supplementary Table S11 (continuation). Results of the parametric one-way ANOVA and Tukey tests for all policy-related variables across countries and spatial levels, including significance (sign, ****: <0.0001, ***: <0.001, **: <0.01, *: <0.05, ns: not significant [>0.05]) overall and cross-groups (Zmb: Zambia, Ecu: Ecuador, Phl: Philippines, Int: International, Nat: National, Reg: Regional, Loc: Local), degrees of freedom (df), sum and mean of squares (SumSq, MeanSq), F-Values, mean differences (diff.) and confidence intervals (lower, upper).

Variable	Parametric one-way ANOVA (transformed variables)						Tukey's HSD (honestly significant difference) test									
		**df**	**SumSq**	**MeanSq**	**F-value**	**Pr(>F)**	**pair**	**diff.**	**lower**	**upper**	**p-adj**	**pair**	**diff.**	**lower**	**upper**	**p-adj**
Financial	**Country**	2	84.05	42.02	71.26	****	**Ecu-Zmb**	-1.4437	-1.7589	-1.1285	****					
	**Residuals**	200	117.95	0.59			**Phl-Zmb**	-0.0999	-0.4053	0.2056	ns	**Loc-Int**	0.4697	-0.2568	1.1962	ns
							**Phl-Ecu**	1.3438	1.0276	1.6601	****	**Reg-Nat**	0.3347	-0.1010	0.7705	ns
	**Spatial level**	3	6.64	2.2142	2.256	ns	**Nat-Int**	0.1851	-0.5071	0.8773	ns	**Loc-Nat**	0.2846	-0.1968	0.7660	ns
	**Residuals**	199	195.36	0.9817			**Reg-Int**	0.5198	-0.1773	1.2170	ns	**Loc-Reg**	-0.0501	-0.5386	0.4383	ns
LandUse Rights	**Country**	2	4.9	2.4522	2.488	ns	**Ecu-Zmb**	0.3808	-0.0266	0.7883	ns					
	**Residuals**	200	197.1	0.9855			**Phl-Zmb**	0.2252	-0.1696	0.6200	ns	**Loc-Int**	0.0031	-0.7347	0.7409	ns
							**Phl-Ecu**	-0.1556	-0.5644	0.2532	ns	**Reg-Nat**	-0.1199	-0.5624	0.3226	ns
	**Spatial level**	3	0.53	0.1757	0.173	ns	**Nat-Int**	0.0846	-0.6183	0.7876	ns	**Loc-Nat**	-0.0816	-0.5704	0.4073	ns
	**Residuals**	199	201.47	1.0124			**Reg-Int**	-0.0352	-0.7432	0.6727	ns	**Loc-Reg**	0.0383	-0.4577	0.5344	ns
Other Policies	**Country**	2	50.41	25.207	33.26	****	**Ecu-Zmb**	1.0852	0.7279	1.4425	****					
	**Residuals**	200	151.59	0.758			**Phl-Zmb**	1.0052	0.6590	1.3515	****	**Loc-Int**	0.0776	-0.6248	0.7800	ns
							**Phl-Ecu**	-0.0800	-0.4385	0.2786	ns	**Reg-Nat**	-0.7252	-1.1464	-0.3039	***
	**Spatial level**	3	19.42	6.472	7.054	***	**Nat-Int**	0.2488	-0.4204	0.9180	ns	**Loc-Nat**	-0.1713	-0.6366	0.2941	ns
	**Residuals**	199	182.58	0.918			**Reg-Int**	-0.4763	-1.1503	0.1976	ns	**Loc-Reg**	0.5539	0.0817	1.0261	*

References

1. Day, M., Gumbo, D., Moombe, K. B., Wijaya, A. & Sunderland, T. *Zambia Country Profile: Monitoring, reporting and verification for REDD+*. vol. 113 (CIFOR, 2014).
2. Nansikombi, H. *et al.* Can de facto governance influence deforestation drivers in the Zambian Miombo? *Forest Policy and Economics* **120**, 102309 (2020).
3. Piotrowski, M. Nearing the tipping point. Drivers of Deforestation in the Amazon Region. (2019).
4. Carandang, A. P. *et al.* Analysis of key drivers of deforestation and forest degradation in the Philippines. *Deutsche Gesellschaft für Internationale Zusammenarbeit (GIZ)* (2013).
5. Hosonuma, N. *et al.* An assessment of deforestation and forest degradation drivers in developing countries. *Environ. Res. Lett.* **7**, 044009 (2012).
6. Geist, H. J. & Lambin, E. F. Proximate Causes and Underlying Driving Forces of Tropical Deforestation: Tropical forests are disappearing as the result of many pressures, both local and regional, acting in various combinations in different geographical locations. *BioScience* **52**, 143–150 (2002).
7. Ferrer Velasco, R. F., Köthke, M., Lippe, M. & Günter, S. Scale and context dependency of deforestation drivers: Insights from spatial econometrics in the tropics. *PLOS ONE* **15**, e0226830 (2020).
8. Curtis, P. G., Slay, C. M., Harris, N. L., Tyukavina, A. & Hansen, M. C. Classifying drivers of global forest loss. *Science* **361**, 1108–1111 (2018).
9. Busch, J. & Ferretti-Gallon, K. What Drives Deforestation and What Stops It? A Meta-Analysis. *Review of Environmental Economics and Policy* **11**, 3–23 (2017).
10. Fischer, R. *et al.* Interplay of governance elements and their effects on deforestation in tropical landscapes: Quantitative insights from Ecuador. *World Development* **148**, 105665 (2021).
11. Lambin, E. F. *et al.* Effectiveness and synergies of policy instruments for land use governance in tropical regions. *Global Environmental Change* **28**, 129–140 (2014).
12. Börner, J., Schulz, D., Wunder, S. & Pfaff, A. The Effectiveness of Forest Conservation Policies and Programs. *Annual Review of Resource Economics* **12**, 45–64 (2020).
